# Bacteriocin Gene-Trait matching across the complete *Lactobacillus* Pan-genome

**DOI:** 10.1038/s41598-017-03339-y

**Published:** 2017-06-14

**Authors:** Fergus W. J. Collins, Paula M. O’Connor, Orla O’Sullivan, Beatriz Gómez-Sala, Mary C. Rea, Colin Hill, R. Paul Ross

**Affiliations:** 1 Teagasc Food Research Centre, Teagasc Moorepark, Fermoy, Cork, Ireland; 2 APC Microbiome Institute, University College Cork, Cork, Ireland; 3 School of Microbiology, University College Cork, Cork, Ireland

## Abstract

Lactobacilli constitute a large genus of Gram-positive lactic acid bacteria which have widespread roles ranging from gut commensals to starters in fermented foods. A combination of *in silico* and laboratory-based screening allowed us to determine the overall bacteriocin producing potential of representative strains of each species of the genus. The genomes of 175 lactobacilli and 38 associated species were screened for the presence of antimicrobial producing genes and combined with screening for antimicrobial activity against a range of indicators. There also appears to be a link between the strains’ environment and bacteriocin production, with those from the animal and human microbiota encoding over twice as many bacteriocins as those from other sources. Five novel bacteriocins were identified belonging to differing bacteriocin classes, including two-peptide bacteriocins (muricidin and acidocin X) and circular bacteriocins (paracyclicin). In addition, there was a clear clustering of helveticin type bacteriolysins in the *Lactobacillus acidophilus* group of species. This combined *in silico* and *in vitro* approach to screening has demonstrated the true diversity and complexity of bacteriocins across the genus. It also highlights their biological importance in terms of communication and competition between closely related strains in diverse complex microbial environments.

## Introduction

Bacteriocins are ribosomally-synthesised antimicrobial peptides which generally act by inducing pore formation or inhibiting cell wall synthesis in target cells^1^. Some bacteriocins such as nisin have found widespread applicability as bio preservatives in food systems where they have been used for decades. Moreover, bacteriocin production can also be a key probiotic trait^2, 3^, and bacteriocins have been suggested as potential alternatives to antibiotics in the future^4^. The *Lactobacillus* genus has a long association with bacteriocin production, with numerous bacteriocins isolated from such species^5–7^. Originally bacteriocin producers were isolated from functional screens against selected target strains, but many studies now rely on prior *in silico* screening, using tools such as BAGEL^8, 9^. BAGEL scans the bacterial genome for putative bacteriocin open reading frames (ORFs) and also analyses surrounding ORFs to search for possible biosynthetic genes, immunity genes and transporters^10^. Whilst the areas of interest identified by BAGEL represent potential bacteriocin operons, this does not always translate into functional bacteriocin production for many reasons including problems with mutation, regulation or target specificity.

There are varying accounts on the extent of bacteriocin production in the environment. While numerous accounts assume ubiquity in production^11, 12^, a definitive analysis has yet to focus on clarifying the actual extent of bacteriocin production. In this study, we elucidate the bacteriocinogenic potential of representative species of the *Lactobacillus* genus and some related genera; i.e. the *Lactobacillus* Genus Complex. Previously Sun *et al*.^13^ analysed the genomes of 175 *Lactobacillus* species and 38 closely related species, carrying out a screen for putative bacteriocin operons using the BAGEL bacteriocin mining tool. Despite no longer formally being considered as bacteriocins, large (>30 kDa) helveticin-like antimicrobial proteins were also included in the study. Based on those results, we analysed strains which were identified as encoding putative bacteriocin operons for *in vitro* production using well diffusion assays (WDAs) and MALDI TOF MS. Well diffusion assays were used to detect antimicrobial production whilst MALDI TOF MS and SDS PAGE were used to identify the masses of the bacteriocins. Peptide masses identified by MS were correlated with the theoretical masses of bacteriocins identified by BAGEL to confirm the identity of the antimicrobial. We reinforced the BAGEL results with BLAST searches for key lantibiotic and sactibiotic enzymes using specific sequences employed in previous studies against this new dataset of *Lactobacillus* genomes^8, 14, 15^. This redundancy allows for a more comprehensive analysis of bacteriocin gene clusters in the sequenced strains.

## Results

### Distribution of Bacteriocin Operons

**Figure 1:**
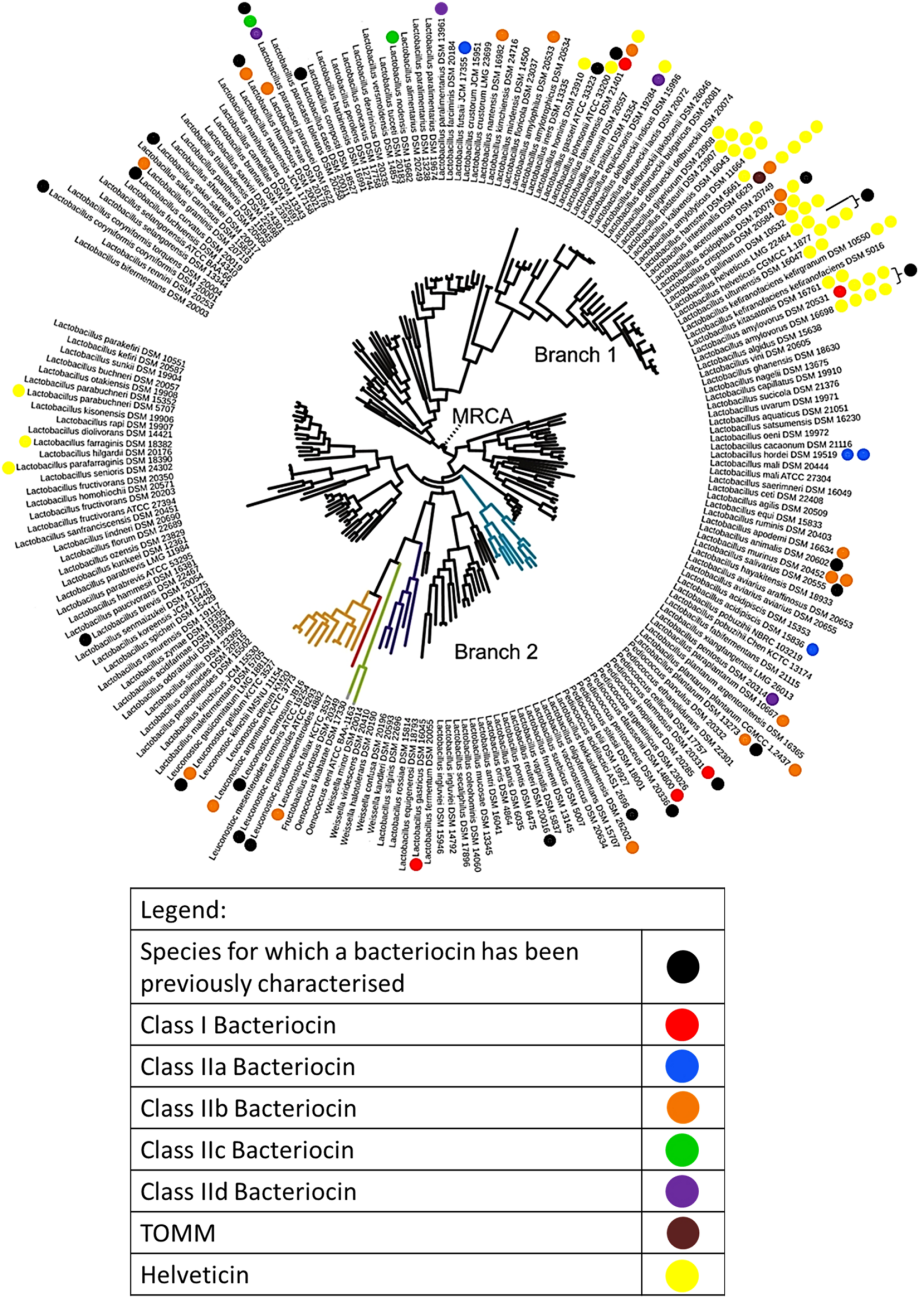
Distribution of complete bacteriocin operons amongst the Lactobacillus Genus Complex (Adapted from Sun *et al*. 12 596).

Several studies have completed bacteriocin screens on diverse and unrelated species of bacteria^8, 16, 17^. The aim of this study was to focus primarily on the lactobacilli and investigate the distribution of bacteriocin genes across this single large important genus. From the information identified by BAGEL, we used a phylogenetic tree to visualise the distribution of bacteriocin operons within the genus (Fig. 1). Historically the *Lactobacillus* genus has a long association with bacteriocin production. While this study focuses on the type strain of each *Lactobacillus* species, Table 1 identifies those bacteriocins which have been previously identified and characterised from all strains in the *Lactobacillus* Genus Complex. In all, 66 bacteriocins have been characterised from lactobacilli previously, which would suggest a high degree of production within the genus. It is notable that the production of these unique bacteriocins is, in fact, restricted to 16 different species.

**Table 1:** Bacteriocins characterised from species within the *Lactobacillus* Genus Complex.

Bacteriocin	Subclass	Producing strain	Origin
**Class I**			
Plantaricin W (α and β)	II	*Lactobacillus plantarum* LMG 2379	Wine
Plantaricin C	II	*L. plantarum* LL441	Cabrales cheese
Lactocin S^a^	II	*L. sakei* L45	Sausages
Pediocin PD-1	II	*Pediococcus damnosus* NCFB1832	Lager Beer
Glycocin F	Glycocin	*L. plantarum* KW30	Fermented corn
**Class II**			
Acidocin A	IIa	*L. acidophilus* TK9201	Fermented milk (starter)
Curvaticin L442	IIa	*L. curvatus* L442	Greek fermented sausage
Curvaticin 13	IIa	*L. curvatus* SB13	Sausages
Sakacin P (variant)^b^	IIa	*L. curvatus* LTH1174	Fermented meat
Plantaricin BM-1	IIa	*L. plantarum* BM-1	Fermented meat
Plantaricin C19	IIa	*L. plantarum* C19	Fermented cucumber
Plantaricin 423	IIa	*L. plantarum* 423	Sorghum (beer)
Sakacin P^c^	IIa	*L. sakei* LTH673	Cured meat
Sakacin A^d^	IIa	*L. sakei* Lb706	Meat
Sakacin G^e^	IIa	*L. sakei* 2512	Food origin
Sakacin X^f^	IIa	*L. sakei* 5	Malt
Bavaricin A	IIa	*L. sakei* MI1401	Sourdough
Bavaricin MN	IIa	*L. sakei* MN	Meat (bovine)
Bacteriocin L-1077	IIa	*L. salivarius* L-1077	Intestine (broilers)
Leucocin A^gh^	IIa	*Leuconostoc geldium* UAL 187	Vacuum-packed meat
Leucocin C	IIa	*Leuc. mesenteroides* TA33a	Spoiled vacuum-packed meat
Leucocin 10C^h^	IIa	*Leuc. mesenteroides* 10	Malted barley
Leucocin 683Y	IIa	*Leuc. mesenteroides* 683	Malted barley
Mesentericin Y105	IIa	*Leuc. mesenteroides* subsp. *mesenteroides* Y105	Goats milk
Pediocin PA-1 (ACH)^i^	IIa	*P. acidilactici* PAC1.0	Meat
Pediocin SA-1	IIa	*P. acidilactici* NRRL B5627	Meat
Penocin A	IIa	*P. pentosaceus* ATCC 25745	Plants
Pediocin SM-1	IIa	*P. pentosaceus* Mees 1934	Meat
Weissellin A	IIa	*Weissella paramesenteroides* DX	Sausage
Lactobin A^j^	IIb	*L. amylovorus* LMG P-13139	Corn liquor
Brevicin 174 (*breB* and *breC*)	IIb	*L. brevis* 174A	Iyokan (fruit)
Lactocin 705 (Lac705α and Lac705β)	IIb	*L. casei* CRL 705	Meat
Acidocin LF221 (LF221A and LF221B)^k^	IIb	*L. gasseri* LF221	Faeces (child)
Gassericin T (GatA and GatX)	IIb	*L. gasseri* SBT2055	Faeces (human)
Lactacin F (LafA and LafX)^l^	IIb	*L. johnsonii* VPI11088	Intestine (human)
**Bacteriocin**	**Type/Subclass**	**Producing strain**	**Origin**
Sakacin T (SakTα and SakTβ)^m^	IIb	*L. sakei* CTC372	Sausages
Plantaricin E/F (PlnE and PlnF)	IIb	*L. plantarum* C11	Fermented cucumber
Plantaricin J/K (PlnJ and PlnK)	IIb	*L. plantarum* C11	Fermented cucumber
Plantaricin S (Plsα and Plsβ)^n^	IIb	*L. plantarum* LPCO10	Green olives
Plantaricin NC8 (PLNC8α and PLNC8β)	IIb	*L. plantarum* NC8	Ensilage
Salivaricin ABP-118 (Abp118α and Abp118β)	IIb	*L. salivarius* UCC118	Intestine (human probiotic)
Salivaricin CLR 1328 (Salα and Salβ)	IIb	*L. salivarius* CLR1328	Vagina (human)
Salivaricin P (Sln1 and Sln2)	IIb	*L. salivarius* DPC6005	Intestine (pig)
Salivaricin T (SalTα and SalTβ)	IIb	*L. salivarius* DPC6488	Intestine (neonate)
Acidocin B	IIc	*L. acidophilus* M46	Food origin
Gassericin A°	IIc	*L. gasseri* LA39	Faeces (child)
Leucocyclicin Q	IIc	*Leuc. mesenteroides* TK41401	Japanese pickles
Acidocin 8912	IId	*L. acidophilus* TK8912	Dairy origin
Brevicin 27	IId	*L. brevis* SB27	Sausages
Lactocin MXJ 32 A	IId	*L. coryniformis* MXJ 32	Fermented vegetables
Curvalicin BAP2	IId	*L. curvatus* CWBI-B28	Meat
Curvaticin FS47	IId	*L. curvatus* FS47	Meat
Sakacin Q (variant)^p^	IId	*L. curvatus* LTH1174	Fermented meat
Bacteriocin SJ2-8	IId	*L. paracasei* BGSJ2-8	Home-made cheese
Paracin C	IId	*L. paracasei* CICC 20241	Probiotic
Plantaricin 1.25 α	IId	*L. plantarum* TMW1.25	Fermented sausages
Plantaricin 1.25 β	IId	*L. plantarum* TMW1.25	Fermented sausages
Plantaricin 149	IId	*L. plantarum* NRIC 149	Pineapple
Plantaricin 163	IId	*L. plantarum* 163	Fermented vegetables
Plantaricin A	IId	*L. plantarum* C11	Fermented cucumber
Plantaricin ASM1	IId	*L. plantarum* A-1	Corn bread
Plantaricin JLA-9	IId	*L. plantarum* JLA-9	Suan-Tsai (Chinese fermented cabbage)
Plantaricin ST31	IId	*L. plantarum* ST31	Sourdough
Sakacin Q^q^	IId	*L. sakei* LTH673	Fermented dry sausage
Salivaricin L	IId	*L. salivarius* DPC6488	Intestine (neonate)
**Bacteriocin**	**Type/ Subclass**	**Producing strain**	**Origin**
Plantaricin Y	IId	*L. plantarum* 510	Koshu vineyard
Rhamnosin A	IId	*L. rhamnosus* 68	Intestinal microbiota (human)
Bactofencin A	IId	*L. salivarius* DPC6502	Intestine (porcine)
Bacteriocin LS2	IId	*L. salivarius* BGHO1	Oral (human)
Leucocin B	IId	*Leuc. mesenteroides* TA33a	Spoiled vacuum-packed meat
Mesentericin 52B^r^	IId	*Leuc. mesenteroides* FR52	Raw Milk
Leucocin N	IId	*Leuc. pseudomesenteroides* QU 15	Nukadoko
Leucocin Q	IId	*Leuc. pseudomesenteroides* QU 15	Nukadoko
Weissellicin 110	IId	*Weissella cibaria* 110	Plaa-Som
Weissellicin L	IId	*W. hellenica* 4–7	Sian-sianzih
Weissellicin M	IId	*W. hellenica* QU 13	Pickel barrel
Weissellicin Y	IId	*W. hellenica* QU 13	Pickel barrel
Lactacin B^s^	—	*L. acidophilus* N2	Food origin
Bacteriocin TSU4	—	*L. animalis* TSU4	Intestine (fish)
Curvalicin BAP3	—	*L. curvatus* CWBI-B28	Meat
Gassericin E	—	*L. gasseri* EV1461	Healthy vagina (human)
Plantacin B	—	*L. plantarum* NCDO1193	Dairy origin
Plantaricin F	—	*L. plantarum* BF001	Spoiled cat fish filets
Plantaricn T	—	*L. plantarum* LPCO10	Green olives
Bacteriocin SMXD51	—	*L. salivarius* SMXD51	Faeces (chicken)
Salivaricin B	—	*L. salivarius* M7	Food origin
**Bacteriolysin**			
Helveticin J		*L. helveticus* NCDO481	Dairy origin

Characterised bacteriocins with identical amino acid sequences: ^a^Sakacin M/lactocin S from *L. sakei* 148. ^b^Variant of sakacin P from *L. curvatus* L442. ^c^Sakacin 674 from *L. sakei* 674. ^d^Curvacin A from *L. curvatu*s LTH1174 and sakacin K from *L. sakei* CTC 494 . ^e^Bacteriocin R1333 from *Lb. sakei* R1333. ^f^Sakacin X from *L. curvatus* 2711 and *L. curvatus* CRL705. ^g^Leucocin A-TA33a from *Leuonostoc mesenteroides* TA33a, and Leucocin B-Ta11a from *Leuc. carnosum* Ta11a. ^h^Leucocin A-4010 and Lecucocin B-4010 from *Leuc. carnosum* 4010. ^i^Also produced by *L. plantarum* WHE92. ^j^Amilovorin L471 from *L. amylovorus* DCE471. ^k^Gassericin K7 (K7A y K7B) from *L. gasseri* K7. ^l^Lactacin F from *L. acidophilus* 30SC. ^m^Sakacin T (SakTα and SakTβ) from *L. sakei* 5, *L. curvatus* 2711 and *L. curvatus* CRL705. ^n^Also produced by *L. pentosus* B96. ^p^Reutericin 6 from *L. reuteri* LA6. ^p^Varient of sakacin Q from *L. curvatu*s L442 and *L. curvatus* CRL705. ^q^Sakacin Q from *L. sakei* Lb674 and sakacin Q from *L. curvatus* CRL705. ^r^Mesentericin B105 from *Leuc. mesenteroides* subsp. *mesenteroides* Y105. ^s^Acidocin J1132 from *L. acidophilus* JCM1132.

Visualisation of the distribution of bacteriocins throughout the *Lactobacillus* Genus Complex shows that there is a clear clustering of helveticin-like operons amongst the *L. acidophilus* branch of species, indicating that such genes have been retained from a common ancestor (Fig. 1). Despite being previously classified as class III bacteriocins, these proteins are now termed bacteriolysins and are considered a distinct group of antimicrobials. Whilst these proteins are ribosomally synthesised, they are much larger than classical bacteriocins (~30 kDa) and are heat labile. Helveticin J is the only member previously characterised^18^, but here we show that these genes are actually highly prevalent in the lactobacilli, with 43 potential homologs identified from 23 strains (for alignment results see Supplementary Figure 1). Of the 18 strains in the *L. acidophilus* group, 36 helveticin homologs were distributed amongst 16 of these strains. While certain strains can encode up to four helveticin homologs, there is insufficient homology between those to suggest recent gene duplications. The high degree of homology (in some cases greater than 99%) between some structural genes encoded by different strains does indicate that horizontal gene transfer of helveticin homologs has occurred; such a mechanism may also explain the presence of these genes in the six strains outside of the *L. acidophilus* group (Fig. 1).

The environment from which these strains have been isolated also seems to correlate with their bacteriocinogenic potential (Supplementary Table 1). For example, of the strains isolated from an animal or human origin 37.5% were identified as encoding a complete bacteriocin or helveticin like operon in BAGEL or BLAST screens (21 of 56 strains). This value for strains isolated from non-animal source (food, plants, environmental and alcohol/wine products) displays an over two-fold reduction at 16.67% (25 of 150 strains). This result suggests that the bacteriocin production may prove to be a competitive advantage for strains from complex environments such as the microbiota of humans and animals.

### Diversity of Bacteriocins Identified

Bacteriocins are a diverse and varied group of antimicrobials, which use different systems for bacteriocin modification, transport and immunity. *In silico* analysis allows us to determine which types of bacteriocins the lactobacilli can synthesise. To analyse the diversity of the bacteriocins encoded by lactobacilli an *in silico* screen was first carried out on the genome of each strain followed by *in vitro* screening of each bacteriocin encoding strain to identify antimicrobial activity against a range of indicators (Table 2). MALDI TOF MS and SDS PAGE allowed us to determine the mass and subsequently the identity of the bacteriocins produced by the strains (Supplementary Figure 2). The bacteriocin classification scheme devised by Cotter *et al*.^1, 4^ was used to distinguish between the different classes of bacteriocins.

**Table 2:** Spectrum of inhibition of bacteriocin producing strains against a range of indicator strains.

Bacteriocin Producers	Strain (DSM)	Activity of Bacteriocin Producers vs. Indicator Organisms*								
		*L. delbrueckii* subsp*. bulgaricus*	*L. delbrueckii* subsp*. lactis*	*L. amylovorus*	*L. casei*	*L. plantarum*	*L. rhamnosus*	*Listeria innocua*	*Enterococcus saccharolyticus*	*E. mundtii*
*L. paralimentarius*	13961				++	+++	++		+	+++
*L. murinus*	20452	+								
*L. hordei*	19519	++	++			++		+++	+++	+++
*L. intestinalis*	6629	+++		+						
*L. paracasei* subsp*. paracasei*	5622	+	+							
*L. acidophilus*	20079	++	++	+					+	
*L. agilis*	20509							+		
*L. crispatus*	20584	++	+	+						
*L. equicursoris*	19284	++								
*L. pentosus*	20314	+								
*L. kalixensis*	16043	+								
*L. amylovorus*	20531	+								
*L. kitasatonis*	16761	++								
*P. damnosus*	20331	+++								
*C. maltaromaticum*	20342	+	+							
*C. maltaromaticum*	20722	++	+					+	++	

Activity of pH neutralised cell free supernatants from bacteriocin producers in agar well diffusion assay. Inhibition of indicators is described in radius (mm) of the zone of inhibition in WDA, scores are as follows: + = 0.5–2 mm, ++ = 2.5–5mm, +++ = >5 mm.

### Class I

Class I bacteriocins are comprised of ribosomally synthesised, post-translationally modified bacteriocins (RiPPs)^4^. Originally restricted to lantibiotics, this class has now been extended to include other post-translationally modified bacteriocins such as sactibiotics.

### Lantibiotics

Lantibiotics are a group of bacteriocins characterised by the presence of lanthionine and methyllanthionine bridges. Here, serine and threonine residues are converted to 2,3-didehydroalanine (Dha) and 2,3-didehydrobutyrine (Dhb), respectively, which then react with the thiol group found in cysteine residues, forming lanthionine or methyllanthionine thioether cross-links^19^. Currently three lantibiotics have been attributed to the *Lactobacillus* genus; lactocin S^20^, plantaricin C^21^ and the two peptide lantibiotic plantaricin W^6^.

The BAGEL screen of the *Lactobacillus* dataset identified three further lactobacilli encoding lantibiotic structural peptides (Table 3, Supplementary Table 2 displays these genes with the associated leader sequence). Of these, potential production was only identified in *L. taiwanensis* DSM 21401 which encodes a type I lantipeptide (a lantibiotic which doesn’t display antimicrobial activity), characterised by the presence of LanB and LanC modification enzymes. What is unusual about this peptide is the fact the structural gene is small compared to other lantipeptides, with the mature peptide predicted to contain only 14 amino acids. Despite a lack of demonstrated antibacterial activity against the range of indicators tested, MALDI TOF MS did identify a mass which correlates with the predicted mass of the mature lantipeptide. The lack of antimicrobial activity may simply imply that the indicator organisms tested were not sensitive, or that the putative lantipeptide has a signalling rather than a bacteriocidal role.

**Table 3:** Potential Lantibiotic/Lantipeptide Structural Peptides.

Species	Strain	Potential Unmodified Lantibiotic/Lantipeptide Sequence
*L. taiwanensis*	DSM 21401	TSTGCCNGPSKLQG
*L. amylovorus*	DSM 20531	AKSYSAYSSCSCVNPPCPIATMD
*L. gastricus*	DSM 16045	GTETAQSTPAISRVTLSIARKSSAKCISWISFSAGGLNSYKSKC
*P. damnosus* (Pediocin PD-1)	DSM 20331	KKIKKSSSGDICTLTSECDHLATWVCC

A further type I lantibiotic operon was identified by BAGEL in the strain *L. amylovorus* DSM 20531. This strain appears to encode a complete lantibiotic operon which contains the required modification enzymes and an ABC transporter. *L. gastricus* DSM 16045 was found to encode a Lan C homolog but a LanB homolog was absent from the operon which is necessary for initial dehydration of serine and threonine residues. The production of either of these bacteriocins was not detected *in vitro*.

Lantibiotic operons were also identified in some of the other genera studied. *Pediococcus damnosus* DSM 20331 was found to encode a class II lantibiotic. This strain has previously been found to produce the partially characterised lantibiotic pediocin PD-1^22^. From genomic data used in this study, the sequence of the pediocin PD-1 gene has now been elucidated, showing a high similarity to the lantibiotic plantaricin C (PlnC)^23^. Due to the similarity between the two bacteriocins, pediocin PD-1 likely shares a common mode of action with PlnC whose activity has been shown to be as a result of the combination of pore formation and inhibition of lipid II synthesis^24^. *P. claussenii* DSM 14800 was also shown to encode pediocin PD-1, however, this strain failed to display bacteriocin production. The *Carnobacterium maltaromaticum* strains DSM 20722 and DSM 20730 were also both found to encode the two-component lantibiotic carnolysin, however the *in vitro* production of this bacteriocin was not seen in either strain^25^.

To supplement the results of BAGEL searches, previous *in silico* lantibiotic screens were repeated on the new *Lactobacillus* dataset. We used the modification enzymes NisC, LtnM1 and VenL as drivers in the BLAST search for novel lantibiotics^8, 14, 15^. *L. gallinarum* DSM 10532, *L. crispatus* DSM 20584 and *P. cellicola* DSM 17757 were all found to harbour a NisC homolog, despite not being identified by BAGEL. However, upon examination of the surrounding genes, no potential structural genes were identified. Strains identified in BLAST searches as encoding LanM homologs had also been identified by BAGEL. No homolog of the novel lanthionine synthase VenL was identified in the BLAST screen.

### Sactibiotics

The sactibiotics are a growing class of bacteriocins characterised by the presence of unusual sulphur to α-carbon linkages. These modifications are carried out by radical S-adenosylmethionine (SAM) proteins which catalyse the formation of these thioether bonds^26, 27^. To analyse the prevalence of potential sactibiotic operons within the lactobacilli, the sequences for the radical SAMs associated with a two-component sactibiotic thuricin CD (TrnC and TrnD) were used as drivers in a BLAST analysis of the genomes available^17, 28^. Only two radical SAMs were found resembling those associated with thuricin CD. *L. mali* DSM 20444 was found to encode one such SAM, however, analysis of the operon failed to identify a potential structural gene. *Kandleria vitulina* DSM 20405 appears to encode a complete sactibiotic operon, encompassing a structural gene, transporter and associated radical SAM, however, no biological activity could be attributed to this strain with the panel of indicators tested. BAGEL further identified two potential sactibiotic related radical SAM proteins in *C. maltaromaticum* DSM 20342 and DSM 20722 but no potential structural gene for these enzymes was apparent.

### TOMMs

Thiazole/oxazole modified microcins (TOMMs) are a class of RiPPs which are now included with the class I bacteriocins. These peptides undergo extensive post-translational modification, with the conversion of cysteine, serine and threonine residues into the corresponding heterocycles; thiazole, oxazole and methyloxazole, respectively^29^. TOMMs exist in gene clusters encoding several factors involved in transport, modification and immunity. Using streptolysin as an example, the modification of the structural peptide is the result of the activity of the SagBCD enzyme complex, encompassing a cyclodehydratase (SagC), a dehydrogenase (SagB) and a docking protein (SagD)^30^. Whilst SagBCD clusters are described as being relatively widespread amongst prokaryotes, no TOMM has yet been identified from a *Lactobacillus* species^30^. In our study *L. crispatus* DSM 20584, *L. intestinalis* DSM 6629 and *Oenococcus kitaharae* DSM 17330 were identified by BAGEL as encoding homologs of the SagBCD gene cluster. Whilst the operons in *O. kitaharae* DSM 17330 and *L. intestinalis* DSM 6629 appear to be complete, the *L. crispatus* DSM 20584 TOMM operon appears to lack a structural gene, however, the structural gene for similar operons has been found to be some distance from the SagBCD homologs previously^31^. Of these three strains, *L. crispatus* DSM 20584 was the only one found to display antimicrobial activity; the source of such activity, however, remains unclear.

### Class II

Class II bacteriocins are small heat stable peptides which are not subject to extensive post translational modification, most of which act to permeabilize the membrane of target cells^1^. This class of bacteriocins is further subdivided based on the structure and activity of the peptides.

### Class IIa

Class IIa or ‘pediocin-like’ bacteriocins display a narrow range of antimicrobial activity, particularly displaying strong anti-listerial activity. Such bacteriocins encompass a highly conserved YGNGV/L N-terminal motif followed by cysteine residues which can form a disulphide bridge. Unlike the N-terminus, the C-terminus is less conserved and is likely involved in membrane insertion and pore formation^32^. These bacteriocins likely act by using the mannose-phosphotransferase system on sensitive cells as a receptor^33^.

Despite having a long association with this class of bacteriocins, surprisingly only 3 *Lactobacillus* strains were found to encode what appear to be complete class IIa bacteriocin operons, containing structural, immunity and transport genes (Table 4a, Supplementary Table 2). Of these, *L. hordei* DSM 19519 displayed bacteriocin production against six of the indicators tested. From MALDI TOF MS and BAGEL results, the production of coagulin was confirmed. This 44 amino acid bacteriocin was originally isolated from *Bacillus coagulans* and closely resembles the bacteriocin pediocin PA-1, differing by a single amino acid due to a N41T substitution^34, 35^. The presence of a further pediocin-like operon was noted within the *L. hordei* genome, encoding a structural peptide displaying 74% amino acid identity to plantaricin 423. Production of this bacteriocin however was not seen.

**Table 4:** Str﻿uctural genes for complete (a) and incomplete (b) Class IIa operons.

Species	Strain	Structural Peptide	Homolog (%Amino Acid Identity)
**(a) Structural Genes for Complete Class IIa Operons**			
*L. hordei*	DSM 19519	KYYGNGVTCGKHSCSVDWGKATTCIINNGAMAWATGGHQGTHKC	Coagulin (100%)
		KYYGNGVSCTKKHGCKVNWGQAFTCSVNRFANFGHGNC	Plantaricin 423 (74%)
*L. acidipiscis*	DSM 15836	KYYGNGLHIPKHGKPYINWGQAIQSIGKISYHGWVNGITSGAAGVGRH	Hiracin JM79 (44%)
*L. futsaii*	JCM 17355	KYYGNGVSCGKHTCKVNWGQAWNESVNRWGNSWVNGLTGLRQH	Plantaricin 423 (57%)
*C. maltaromaticum*	DSM 20722	AISYGNGVYCNKEKCWVNKAENKQAITGIVIGGWASSLAGMGH	Carnobacteriocin cbn BM1 (100%)
		VYYGNGVSCSKTKCSVNWGQAFQERYTAGINSFVSGVASGAGSIGRRP	Carnobacteriocin cbn B2 (98%)
**(b) Structural Genes for Incomplete Class IIa Operons**			
*L. agilis*	DSM 20509	SRYYGNGITCGKHKCTVNWGQAWTCGVNRLANFGHGNC	Plantaricin 423 (73%)
*L. aquaticus*	DSM 21051	KNYGNGVYCTKKHGYKVDWGQAWSIIGNNSAANSTTRGAAGWKSK	Avicin A (74%)
*L. rennini*	DSM 20253	KYYGNGVSCSKHSCSVDWGKALTCTINNGAMAWTTGGHQGNHKC	Pediocin Ach/PA-1 (89%)
*L. ruminis*	DSM 20403	KYYGNGVYCGKHKCRVDWGQAWGCSVNRWGAAVGTGGKATIGHC	Pediocin Ach/PA-1 (55%)
*P. pentosaceus*	DSM 20336	KYYGNGLYCGKHSCSVDWGKATTCIINNGAMAWATGGHQGTHKC	Pediocin Ach/PA-1 (93%)
*C. maltaromaticum*	DSM 20342	AISYGNGVYCNKEKCWVNKAENKQAITGIVIGGWASSLAGMGH	Carnobacteriocin cbn BM1 (100%)

Numerous lactobacilli identified in this study were found to carry partial pediocin-like operons, often containing the bacteriocin structural gene and associated immunity protein but lacking the appropriate transporters (Table 4b, Supplementary Table 2). One potential explanation is that when a strain acquired the gene for pediocin resistance that the neighbouring small bacteriocin structural gene was also transferred, whilst the larger transporters were not.

Although not included in the *Lactobacillaceae* family, several *Carnobacterium* strains were included in the genomic study carried out by Sun *et al*.^13^. Numerous bacteriocins have been attributed to this genus previously^25, 36^. While the source of antimicrobial activity from *C. maltaromaticum* DSM20342 is unclear, C*. maltaromaticum* DSM 20722 was found to produce the class IIa bacteriocin cbnB2 and cbnBM1, the class IId bacteriocin cbnX was also produced by the strain^25^. CbnB2 contains an N2Y mutation which was also previously seen by Tulini *et al*.^25^.

### Class IIb

Class IIb are comprised of unmodified two peptide bacteriocins, whose activity is dependent on the synergistic activity of both peptides which interact to form a single antimicrobial unit^37^. These bacteriocins are likely to act by forming membrane spanning pores which result in the leakage of small molecules from the cell. Such bacteriocins tend to contain conserved GxxxG or AxxxA motifs which are responsible for close helix interactions between each bacteriocin peptide^37^. A wide range of class IIb bacteriocins were identified by BAGEL in this study (Table 5, Supplementary Table 2).

**Table 5:** Potential Class IIb Structural Genes.

Species	Strain	Structural Peptide		
*L. murinus*	DSM 20452	YNRLAGQIGHYTGKAVIVGATVLGIASLF	Produced *in vitro*	(Muricidin)
		KRGLGYHIVDAVVSFGKGFLDAF		
		YDIEKALWGGYGYQLGWRNKWNLSHRYFKI		
		GVPGWYYGMLWKIGVSGYKHRKDIMNGFDRGFNNYPK		
*L. acidophilus*	DSM 20079	SNNIFWTRVGVGWAAEARCMIKPSLGNWTTKAVSCGAKGLYAAVRG	Produced *in vitro*	(Acidocin X)
		VAPIVYPIAGYVMKQMFEHSDQIIKGFKRGWKKYK		
*L. taiwanensis*	DSM 21401	NRWGDTVLSAASGAGTGIKACKSFGPWGMAICGSNRRLFWLYS		
		RNNWQTNVGGAVGSAMIGATVGGTICGPACAVAGAHYLPILWTGVTAATGGFGKIRK		
*L. crispatus*	DSM 20584	NRWTNAYSAALGCAVPGVKYGKKLGGVWGAVIGGVGGAAVCGLAGYVRKG		
		SKGKGRNNWAGNTIGIVSSAATGAALGSAICGPGCGFVGAHWGAVGWTAVASFSGAFGKIRK		
*L. nantensis*	DSM 16982	SFKGFVQGFINGLTGKKH		
		KGPWNYKTGYNLGKWISKRF		
*L. apodemi*	DSM 16634	YDIEKALWKGYGYQLGWRSKWNLSHRYFKI		
		GVPGWYYSMLWKIGVSGYKHRKDIMSGFDKGFNNYPK		
*L. plantarum*	DSM 13273	RRSRKNGIGYAIGYAFGAVERAVLGGSRDYNK		
		GAWKNFWSSLRKGFYDGEAGRAIRR		
		FNRGGYNFGKSVRHVVDAIGSVAGIRGILKSIR		
		VFHAYSARGVRNNYKSAVGPADWVISAVRGFIHG		
*L. plantarum* subsp*. plantarum*	CGMCC 1.2437	RRSRKNGIGYAIGYAFGAVERAVLGGSRDYNK		
		GAWKNFWSSLRKGFYDGEAGRAIRR		
		FNRGGYNFGKSVRHVVDAIGSVAGIRGILKSIR		
		VFHAYSARGVRNNYKSAVGPADWVISAVRGFIHG		
*L. paraplantarum*	DSM 10667	FNRGGYNFGKSVRHVVDAIGSVAGIRGILKSIR		
		VFHAYSARGVRNNYKSAVGPADWVISAVRGFIHG		
*L. intestinalis*	DSM 6629	RHSVPYSYGYQSGRGFKGAAAAYNIIKTVASFFE		
		KRKKHHPWYWSIQEFGRGFLAGLASKYNL		
*L. rhamnosus*	DSM 20021	IGPLAIPVAAILGFLATDAWSHADELVAGVKQGWERS		
		DNGNLWTFIGKAIGSTARSWAEGAMFAPAIGPAKEIVDKLNGN		
*L. zeae*	DSM 20178	NAWGNAVNGALNGAATGARFGKNLGPWGMIGGMALGAGIGGYFGYNG		
		RNTWQQNVSGVAGAAAGGAALGAVVGGPAGAFLGAHYGPILWTAVTGFTGGF		
*Leuc. fallax*	KCTC 3537	CPLLPIVVTVAASGAHFVAKDGWNHLDQIRSGWRKSGNSKW		
		STDGSWEDFGAGLHKTVNTVIYAGTTVARAHTRSHQRCFTGNKW		

*L. murinus* DSM 20452 was one of the strains which demonstrated bacteriocin production. MALDI TOF MS identified masses which correlate with a two-peptide bacteriocin identified by BAGEL (muricidin). Both peptides of muricidin display homology to the class IIa bacteriocin plantaricin S, with the α peptide displaying 41% amino acid identity to pln Sα and the β peptide 48% to pln Sβ. The β peptide found here however lacks the AxxxA motif found in pln Sβ, a sequence which has been shown to be important for helix-helix interactions in pln S^38^.

Another potential two-peptide bacteriocin (acidocin X) was also identified from *L. acidophilus* DSM 20079. Correlation between the bacteriocins identified by BAGEL and the results of MALDI TOF MS led to the identification of two, bacteriocin like, peptides. The first of these was a 35 amino acid peptide displaying 53% identity with the enterocin X β peptide. The second peptide was not identified in BAGEL and was found by manual analysis of the bacteriocin operon, this displays 25% identity to the enterocin X α peptide.

### Class IIc

Class IIc bacteriocins are also known as circular bacteriocins due to the covalent linkage of the N- to C-termini. The compact circular structure of these bacteriocins can contribute to their temperature and pH stability^39^. These circular bacteriocins permeabilize the target cell membrane, resulting in a loss of membrane potential which leads to cell death^40^. Despite having similar modes of action, this class of bacteriocins are further broken down into two subgroups, based on the isoelectric point of the peptides and the conservation seen amongst the groups^41^. Currently, there are two examples of class IIc bacteriocins produced from lactobacilli, both of which belong to subgroup II. Originally identified as two separate class IIc bacteriocins, Gassericin A (*L. gasseri* LA39) and reutericin 6 (*L. reuteri* LA6) have now been shown to be identical^42, 43^. Acidocin B (*L. acidophilus* M46), originally thought to be linear, has also been recently reclassified as a circular bacteriocin. *Leuconostoc mesenteroides* TK41401 has also been shown to produce leucocyclicin Q, a subgroup I circular bacteriocin.

From the analysis carried out in this study, *L. paracasei* subsp. *paracasei* DSM 5622 was found to produce a potential class IIc bacteriocin (paracyclicin), with a structural gene displaying 64% amino acid identity to butyrivibriocin AR10^44^. The operon contains a putative ABC permease, ATPase and a protein belonging to the DUF 95 protein family, all of which have been associated with the gene clusters of circular bacteriocins^39^. Upon purification of the bacterial supernatant, a mass of 5905.75 Da was identified as the causative agent of antimicrobial activity. This mass correlates closely with the predicted mass of the mature bacteriocin structural peptide which is calculated as 5906.87 Da. It is clear that paracyclicin belongs to the subgroup II circular bacteriocins, due to a high level of conservation found within the group (Table 6). Despite this conservation, this novel bacteriocin does display variation in certain conserved regions which is not seen in the rest of the class. *L. nodensis* DSM 19682 was also found to encode one such potential bacteriocin, however, no antimicrobial activity was observed with this strain.

**Table 6:** Alignment of Class IIc Subgroup II Bacteriocins.

Bacteriocin	Structural Peptide
Gassericin A	IYWIADQFGIHLATGTARKLLDAMASGASLGTAFAAILGVTLPAWALAAAGALGATAA
Acidocin B	IYWIADQFGIHLATGTARKLLDAVASGASLGTAFAAILGVTLPAWALAAAGALGATAA
Butyrivibriocin AR10	IYFIADKMGIQLAPAWYQDIVNWVSAGGTLTTGFAIIVGVTVPAWIAEAAAAFGIASA
*L. paracesei* subsp. *paracasei* DSM 5622 (Paracyclicin)	IYFIANKLGIHLAPGWYQDMVNYVSAGGSLAGAFSVVAGVTLPAWIVPIATAFGAVSA
*L. nodensis* DSM 19682	-IWIAGLFGIHLDNSLESKLVSGILNGGSAAGVFAAMLGITLPAWAAAAATAMGATAA
	:**:**: *..::.:*.:*::*:*:*****:*.:*

* = Positions with a single conserved residue. ^:^ = Conservation between groups with strongly similar properties, scoring >0.5 in the Gonnet PAM 250 matrix. ^.^ = Conservation between groups with weakly similar properties, scoring ≤0.5 in the Gonnet PAM 250 matrix.

### Class IId

Class IId bacteriocins are single peptide, linear bacteriocins which do not display homology to the pediocin like bacteriocins^4^. This class of bacteriocins displays a high degree of diversity and numerous class IId bacteriocins have been characterised from lactobacilli previously (Table 1). *In silico* analysis of the *Lactobacillus* dataset identified numerous novel structural genes (Table 7, Supplementary Table 2) with several shown to be produced.

**Table 7:** ﻿Potential Class IId﻿ structural proteins (a) and Class IId lactococcin 972 homologs (b).

Species	Strain	Structural Protein
**(a) Potential Class IId Structural proteins**		
*L. paralimentarius*	DSM 13961	NFFGGSNGYSWRDKKGHWHYTVTSGVSSTVAQIIGNGWGSAGAPGVGQR
*L. pentosus*	DSM 20314	KSNTYSLQMGSVVRTATKIFKKMEW
*L. hokkaidonensis*	DSM 26202	VTLSVATHSKNGLKKFFKWVRKL
*L. xiangfangensis*	LMG 26013	KLVKLYTAEPYTFYRDTRTKKIVMRQTTGYSAHLQHVIADGWVRSAHL
*L. paracasei*	DSM 5622	DSIRDVSPTFNKIRRWFDGLFK
*L. murinus*	DSM 20452	YDIEKALWGGYGYQLGWRNKWNLSHRYFKI
*Leuc. kimchii*	IMSNU 11154	KSFWSWASDASSWLSGPQQPNSPLLKKKR
*Leuc. geldium*	KCTC 3527	KRVYIPNGNGAWLDSNTGKGGVDWNVAVPALGSIMVNGWAQNGPLAHLHP
**(b) Potential Class IId Lactococcin 972 Homologs**		
*L. equicursoris* (equicursorin)	DSM 19284	GGTWNYGVGSKYVWSYYSHNSKTHKASVEGKYYVTSGWIKEKTQARASAAKAAAGNQSYYDVK
*L. amylophilus*	DSM 20533	GGTWNYGVGLTGTFGYSDYLHNSKTHSASVGRTKSDCNKVTKTKGVWAQSKYTKIPPTGLNYWWSVS
*L. graminis*	DSM 20719	GGTWYSGFSGTKVYSQYYHGSKKHSATAKNGWGAGVRNTQKAGIWAYSSVNSTLTGNKTYWAVY
*L. hamsteri*	DSM 5661	GGVWNYGVGKKYVWSYYSHHRLTHKSSVEGKYYSSSGWVSPGTEARASAEKAQHGNKSYFDVE
*Leuc. argentinum*	KCTC 3773	GGDWRHGVGSYYVWSYYFHNYRNHSSSVSGQYFASSGRTSPGYDAQASAPKSLFGNKAYYDFW

*L. paralimentarius* DSM 13961 was one such strain to display the production of a class IId bacteriocin (paralimenterocin). The paralimenterocin structural gene identified encodes a 44 amino acid single peptide bacteriocin whose closest homolog appears to be the relatively uncharacterised bacteriocin BacSJ2-8 to which it has 77% identity^45^. The mode of action of both of these bacteriocins remains unclear.

*L. equicursoris* DSM 19284 is also highly likely to produce a novel class IId bacteriocin (equicursorin). The strain displayed antimicrobial production upon analysis, but MALDI TOF MS did not identify an associated mass. *In silico* BAGEL analysis identified three putative bacteriocin operons, two of which encoded larger bacteriolysins of approximately 30 kDa, the remaining operon encodes a homolog of lactococcin 972. SDS PAGE analysis of the concentrated culture supernatant identified a mass between the 5 kDa and 10 kDa markers which displayed antimicrobial activity once overlaid with *L. delbrueckii* subsp. *bulgaricus* LMG 6901 (Supplementary Figure 2). This mass correlates well with the predicted mass (approximately 7 kDa) of the lactococcin 972 homolog ‘equicursorin’. Lactococcin 972 is unique with respect to its activity in comparison to other class II bacteriocins. These bacteriocins do not induce pore formation in the cells but instead act by binding to lipid II and inhibiting septum formation. Lactococcin 972 is also unusual in that it’s biologically active form is as a homodimer^46, 47^. Given that only two such bacteriocins have been identified, it was surprising that four further lactococcin 972-like operons were identified in th﻿e genomic dataset screened in this study (Table 7(b), Supplementary Table 2). An *in silico* screen carried out by Letzel *et al*.^48^ identified 9 further Lactococcin 972 operons in anaerobic bacteria, thus due to the expansion of this group, these bacteriocins may warrant a separate classification, given their unique mode of action when compared to other class II bacteriocins.

### Bacteriolysins (Formerly Class III Bacteriocins)

In the *Lactobacillus* dataset, a number of homologs of the bacteriolysin helveticin^18^ were found to be encoded, with several displaying *in vitro* antimicrobial activity. The approximate size of these proteins was determined using SDS PAGE overlay assays, as MALDI TOF MS was not used to determine the size of these larger proteins. Several strains encoded numerous helveticin homologs, however, SDS PAGE overlays were not able to identify which of these homologs was actually produced as all had masses of approximately 37 kDa (Supplementary Figure 3).

*L. intestinalis* DSM 6629 was shown to produce one of these helveticin homologs, with four potential structural genes found within the genome ranging from 38% to 67% amino acid identity to helveticin J. *L. kitasatonis* DSM16761 also produced a helveticin like peptide, the strain encodes two such proteins displaying 35% and 41% identity to helveticin J. Two *L. amylovorus* strains (DSM 16698 and DSM 20531) were shown to produce a helveticin homolog. *L amylovovrus* DSM 16698 encodes four of such proteins, whilst *L. amylovorus* DSM 20531 encodes three. Both share a single identical helveticin homolog but it is unclear whether this is the protein produced by both strains. *L. kalixensis* DSM 16043 also produces a helveticin-like protein, with 3 homologs encoded within the genome displaying, 34%, 49% and 50% amino acid identity to helveticin J.

BAGEL also identified a helveticin homolog (77% identity to helveticin J) from *L. crispatus* DSM20584. Interestingly, analysis of the results of an exoproteomic study carried out by Johnson *et al*.^49^ identified the secretion of this protein previously. The antimicrobial activity of the strain in this study was determined to be due to a small peptide by an SDS PAGE overlay assay, this is most likely a lactacin F homolog^50^ or else a novel TOMM like peptide.

## Discussion

This study gives the first complete assessment of bacteriocin production across the *Lactobacillus* Genus Complex, combining both *in silico* and laboratory based screening methods. This combination of approaches allows for a more representative estimation of bacteriocin production to be calculated. Well-diffusion assays and MALDI TOF MS allows for the confirmation of *in vitro* bacteriocin production by cells. Bacteriocin production however can be a highly regulated process, with strains requiring specific conditions and environments to induce production of these antimicrobials^51, 52^. Such regulations would make it extremely difficult to identify the bacteriocins found here using *in silico* screens if we were to rely on *in vitro* screening methods alone. Thus, the use of BAGEL and BLAST bacteriocin screens allows us to identify these bacteriocin operons from the *Lactobacillus* Genus Complex without the shortcomings and restrictions of laboratory based screens.

*In silico* analysis has allowed us to determine the overall bacteriocinogenic potential of the *Lactobacillus* genus. Of the 213 strains analysed, 51 were identified by BAGEL or in BLAST screens as harbouring what appears to be a complete bacteriocin or helveticin like operon, a prevalence of 23.94%. If we focus on the lactobacilli, of the 175 strains analysed only 25 were found to encode bacteriocin operons (14%). If helveticin operons and those of previously characterised bacteriocins are included, of the *Lactobacillus* species analysed 30% were found to encode at least one antimicrobial. This figure of 30% is surprisingly high given that lactobacilli are not associated with the production of more traditional antibiotics formed by non-ribosomal peptide synthetases (NRPS) and polyketide synthases (PKS). Given the extent of bacteriocin production within the genus, the production of antimicrobials by these means may be unnecessary, especially given the size of such NRPS and PKS operons and the subsequent energy it would take to produce them. Thus, bacteriocin production may supplant the need for NRPS and PKS enzyme complexes in certain genera.

There was a high degree of novelty within the bacteriocins identified by BAGEL in this study and of all the structural genes identified, 73% had not previously been characterised. Screening of these strains identified five novel functional bacteriocins (muricidin, acidocin X, paracyclicin, paralimenterocin and equicursorin) from a range of bacteriocin classes. In addition, five novel producers of helveticin-like peptides were also identified. The abundance of homologs of helveticin-like bacteriolysins encoded by lactobacilli is surprising given how little these proteins have been characterised to date. The observation that most strains in the *L. acidophilus* group encode helveticins with significant homology suggests that this trait was derived from a common ancestor and then disseminated by horizontal transfer. Apart from narrow spectrum antimicrobial activity, no other function has been ascribed to these proteins. The role these proteins play in the life cycle of this narrow branch of strains warrants further study.

The variety and distribution of bacteriocins throughout the genus is interesting when compared to the results of other *in silico* screens which were carried out. Letzel *et al*.^48^ used BAGEL and other tools to screen the genomes of 211 anaerobes for bacteriocin encoding genes (no lactobacilli were included in the screen, and helveticin like proteins were excluded). Of these 211 strains, just over 25% were found to encode a bacteriocin like peptide. Thus despite the differences in the make-up of the datasets, there is a similar level of bacteriocin encoding genes found in both groups. While the overall levels may be similar, the diversity of the bacteriocins encoded differs greatly. Of the bacteriocins encoded in the anaerobic dataset, 78% were found to be class I modified bacteriocins, while in the *Lactobacillus* Genus Complex this value is only 17%. One similarity between these sets of results, however, is the presence of lactococcin 972 like bacteriocins. 9 novel homologs were identified in the anaerobic bacteria, this result taken with the number of novel homologs identified from the lactobacilli suggests that this group of unique bacteriocins merit their own class of bacteriocins in the future given their unique mode of action and increasing prevalence.

In a bioinformatic screen of *Bacillus* species for bacteriocin operons^53^, the overall level of bacteriocins encoded by such strains was much higher, with 583 putative bacteriocin operons encoded in the genomes of 328 strains. 89% of these strains, covering 50 different species encode a bacteriocin, a much higher level than seen in the anaerobic bacteria and the lactobacilli. The diversity of encoded bacteriocins again differs to that of the lactobacilli with 66% of operons identified here encoding class I bacteriocins. This difference suggests that there is not an even distribution in the types of bacteriocins across genera, with the lactobacilli in particular relying on the production of class II bacteriocins in comparison to other groups. A similar high prevalence of bacteriocin operons can be found in the cyanobacteria, with 145 putative bacteriocin gene clusters being identified in 43 of the 58 complete and partial genomes screened^54^. It must be remembered, however, that in both studies these operons were not manually analysed so, in reality, overall levels may be lower.

The inter-species diversity of bacteriocin production can be seen in a screen carried out by Liu *et al*.^55^ whereby, the genomes of 169 *Streptococcus mutans* strains were screened by BAGEL for bacteriocin operons. 211 bacteriocin operons were found distributed amongst 157 strains, of which 32 were lantibiotic operons. These results show that despite carrying out a comprehensive analysis of bacteriocin production in lactobacilli, a high level of diversity within each species can still result in novel bacteriocins being identified.

The environment from which strains are isolated may also influence their bacteriocinogenic potential. 37.5% of strains isolated from human and animal microbiomes encoded bacteriocins or bacteriolysins, this is over twice the value for strains isolated from food, wine and beer, plants and the environment at 16.67%. The microbiota of animals is a complex community with microbes under constant competition for nutrients and resources^56^. Bacteriocin production can provide a competitive advantage for strains, allowing them to inhibit sensitive strains thus reducing competition and allowing them to establish themselves in these environments^2, 3, 57^. This may suggest why a greater proportion of lactobacilli from these environments encode bacteriocins. Environments such as fermented foods would provide a much narrower niche for the growth of microbes. Less competition here may negate the need for these bacteria to expend energy on bacteriocin production.

Given the association of lactobacilli with probiotics and food production, the knowledge of their potential to produce antimicrobials is of great value^58^. Bacteriocin production may increase their ability to establish themselves in a community such as the gut, or provide a natural mechanism to inhibit the growth of food spoilage microorganisms^3, 59^. Thus bacteriocin production can prove a useful trait for an industrially important group of bacteria. Previously, the isolation of bacteriocins from lactobacilli relied on intensive laboratory screens of individual cultures. The use of tools such a BAGEL and BLAST however now allow for the rapid identification of bacteriocin operons within strains, and with the increasing availability of genomic data, these tools are becoming more relevant.

## Materials and Methods

### Bacteriocin Identification

The bacteriocin mining tool BAGEL2 was used to identify putative bacteriocin operons^10^ and the genome visualisation tool ARTEMIS was subsequently used for manual analysis of the bacterial genomes^60^. To determine the degree of novelty in the bacteriocins identified by BAGEL2, BLASTP searches were done for each putative bacteriocin peptide against those identified in the BAGEL screen. The levels of identity described in this study are derived from Clustal Omega. For bacteriocin analysis using specific “driver” sequences, the BLASTP program was used using default parameters. The driver sequences used were NisC (GenBank Accession no. CAA79470.1), LtnM1 (GenBank Accession no. NP_047321.1), VenL (GenBank Accession no. AEA03262.1), TrnC and TrnD from *Bacillus thuringiensis* DPC 6431.

### Bacterial strains

The bacterial strains screened for bacteriocin production and the conditions for growth are listed in Supplementary Table 3. Anaerocult A gas packs (Merck, Darmstadt, Germany) were used to generate anaerobic conditions.

### Bacteriocin Assays

Bacteriocin activity was analysed via well diffusion assays against the indicator organisms listed in Supplementary Table 4. Briefly, each strain screened was grown in broth under the appropriate conditions. The cell free supernatant of each culture was prepared by centrifugation of the fully grown culture at 4000 RCF for 20 minutes, the pH was adjusted to pH 7 using sodium hydroxide to negate any antimicrobial activity which may be caused by the acidity of the cell free supernatants. 50 μl of an overnight culture of each indicator was then added to 20 ml of the appropriate media containing 1.5% agar. Plates were allowed cool and the 7 mm wide wells were bored into the agar. 50 μl of the cell free supernatant of the strains being tested was then placed in a well. These indicator plates were refrigerated for two hours prior to incubation.

### Mass Spectrometry (MS)

MALDI TOF colony mass spectroscopy was carried out on each of the strains as described by Field *et al*.^61^ to identify masses of putative bacteriocins. Here colonies were first mixed with a 70% propan-2-ol 0.1% TFA solution to elute bacteriocin from the cell. Following centrifugation, the subsequent supernatant was spotted on the target pre-coated with CHCA matrix solution. A further layer of matrix solution was then added on top of this supernatant. An Axima TOF^2^ MALDI TOF mass spectrometer (Shimadzu Biotech, Manchester, UK) was used to identify the peptide masses using positive-ion reflectron mode.

### Sodium Dodecyl Sulfate-Polyacrylamide Gel Electrophoresis (SDS-PAGE)

SDS PAGE was used for the identification of higher molecular weight antimicrobial proteins (bacteriolysins). Cultures were grown overnight in broth and the cell free supernatants were prepared as described above. The proteins from the bacterial supernatant were precipitated by the addition of ammonium sulphate salts up to a concentration of 50%. The precipitate was collected by centrifugation and resuspended in water. Supernatants were then incubated with TruPAGE^TM^ LDS sample buffer (Sigma-Aldrich, Wicklow, Ireland) for 10 minutes at 70°C. Samples were run on 12% acrylamide gels at 30 mA, together with Precision Plus Protein™ Dual Xtra prestained protein standards (Bio-Rad, Hertfordshire, UK) which were used to estimate molecular mass with a range of 2–250 kDa. The completed gels were divided in two, one half was stained using the EZBlue^TM^ staining reagent (Sigma-Aldrich). The other half was washed with 1% tween-80 (Sigma-Aldrich) for 45 minutes, followed by three 5 minute washes in distilled water. This gel was overlaid with soft MRS agar (0.8% agar), seeded with 0.25% of an overnight culture of *L. delbrueckii* subsp. *bulgaricus* LMG 6901. The plate was incubated overnight to determine the mass of any antimicrobial proteins produced.

### Bacteriocin Purification

#### Carnobacteriocins CbnB2, CbnBM1 and CbnX

*Carnobacterium maltaromaticum* DSM 20722 was grown overnight in TSA broth, 100 ml of the supernatant was passed through a 5 g, 20 ml Strata C18-E solid-phase extraction (SPE) column (Phenomenex, Cheshire, UK). The column was washed with 20 ml of 30% ethanol and 20 ml of 70% 2-propanol (IPA) 0.1% TFA. The 70% IPA eluent was concentrated and applied to a Semi Prep Proteo Jupiter RP-HPLC column (10 × 250 mm, 90 Å, 4 µ) (Phenomenex, Cheshire, UK) running a 20–55% gradient whereby buffer B was 90% acetonitrile. MALDI TOF MS was carried out on fractions to identify the presence of the peptides of interest.

#### Paracyclicin

*L. paracasei* subsp. *paracasei* DSM 5622 was grown overnight in MRS broth. Culture supernatant was passed through a column containing 60 g Amberlite XAD beads and washed with 400 ml of 50% ethanol and the antimicrobial peptide eluted with 400 ml of 70% IPA 0.1% TFA. The IPA was removed and the eluent passed through a 5 g, 20 ml C18 SPE column pre-equilibrated with methanol and water. The column was washed with 30 ml of 50% ethanol and activity eluted with 30 ml of IPA. The IPA was removed from the C18 SPE IPA eluent and the sample applied to a semi preparative Vydac C4 Mass Spec (10 × 250 mm, 300 Å, 5 µ) RP-HPLC column (Grace, Columbia, USA) running an acetonitrile and propan-2-ol gradient described as follows: 5–55% buffer B and 0–5% buffer C over 25 minutes followed by and 55–19% buffer B and 5–81% buffer C over 60 minutes, 19–5% buffer B and 81–95% buffer C over 5 minutes where buffer A is Milli Q water containing 0.1% TFA, buffer B is 90% acetonitrile 0.1% TFA and buffer C is 90% propan-2-ol 0.1% TFA. Eluent was monitored at 214 nm and fractions were collected at 1 minute intervals. Fractions were assayed using well diffusion assays against *L. delbrueckii* subsp. *bulgaricus* LMG 6901. MALDI TOF MS was used to determine the mass of the antimicrobial peptide.

## Electronic supplementary material

Supplementary Information
