# Supplementary material for: Bacteriocin Gene-Trait matching across the complete Lactobacillus Pan-genome
Source: Sci Rep. 2017 Jun 14;7:3481. doi: 10.1038/s41598-017-03339-y (PMC5471241; doi:10.1038/s41598-017-03339-y)

# Supplementary Information

# Bacteriocin Gene-Trait matching across the complete *Lactobacillus* Pan-genome.

Fergus W. J. Collins[1,2,3], Paula M. O'Connor[1,2], Orla O'Sullivan[1,2], Beatriz Gomez-Sala[1], Mary C. Rea[1,2], Colin Hill[2,3] and R. Paul Ross[1,2,3*]

[1]Teagasc Food Research Centre, Teagasc Moorepark, Fermoy, Cork, Ireland, [2]APC Microbiome Institute, University College Cork, Ireland, [3]Department of Microbiology, University College Cork, Ireland

* Correspondence: p.ross@ucc.ie

Address: College of Science, Engineering and Food Science (SEFS), University College Cork, Ireland

Phone: 00353214903760

**Supplementary Table 1.** Source of Strains

| Species Name | StrainID | Source | Antimicrobial Encoded |
|---|---|---|---|
| *Lactobacillus agilis* | DSM-20509 | Environment | |
| *Lactobacillus aquaticus* | DSM-21051 | Environment | |
| *Lactobacillus concavus* | DSM-17758 | Environment | |
| *Lactobacillus coryniformis torquens* | DSM-20004 | Environment | |
| *Lactobacillus paracollinoides* | DSM-15502 | Environment | |
| *Lactobacillus sharpeae* | DSM-20505 | Environment | |
| *Weissella kandleri* | DSM-20593 | Environment | |
| *Carnobacterium divergens* | DSM-20623 | Food | |
| *Carnobacterium maltaromaticum* | DSM-20342 | Food | |
| *Carnobacterium maltaromaticum* | DSM-20722 | Food | Yes |
| *Lactobacillus acetotolerans* | DSM-20749 | Food | |
| *Lactobacillus acidifarinae* | DSM-19394 | Food | |
| *Lactobacillus acidipiscis* | DSM-15836 | Food | Yes |
| *Lactobacillus acidipiscis* | DSM-15353 | Food | |
| *Lactobacillus algidus* | DSM-15638 | Food | |
| *Lactobacillus alimentarius* | DSM-20249 | Food | |
| *Lactobacillus bifermentans* | DSM-20003 | Food | |
| *Lactobacillus capillatus* | DSM-19910 | Food | |
| *Lactobacillus casei* | DSM-20011 | Food | |
| *Lactobacillus collinoides* | DSM-20515 | Food | |
| *Lactobacillus crustorum* | LMG-23699 | Food | |
| *Lactobacillus crustorum* | JCM-15951 | Food | |
| *Lactobacillus curvatus* | DSM-20019 | Food | |
| *Lactobacillus delbrueckii bulgaricus* | DSM-20081 | Food | |
| *Lactobacillus delbrueckii delbrueckii* | DSM-20074 | Food | |
| *Lactobacillus delbrueckii indicus* | DSM-15996 | Food | |
| *Lactobacillus delbrueckii lactis* | DSM-20072 | Food | |
| *Lactobacillus farciminis* | DSM-20184 | Food | |
| *Lactobacillus frumenti* | DSM-13145 | Food | |
| *Lactobacillus fuchuensis* | DSM-14340 | Food | |
| *Lactobacillus futsaii* | JCM-17355 | Food | Yes |
| *Lactobacillus hammesii* | DSM-16381 | Food | |
| *Lactobacillus harbinensis* | DSM-16991 | Food | |
| *Lactobacillus helveticus* | CGMCC-1.1877 | Food | Yes |
| *Lactobacillus kefiranofaciens kefiranofaciens* | DSM-5016 | Food | |
| *Lactobacillus kefiranofaciens kefirgranum* | DSM-10550 | Food | Yes |
| *Lactobacillus kefiri* | DSM-20587 | Food | |
| *Lactobacillus kimchicus* | JCM-15530 | Food | |
| *Lactobacillus kimchiensis* | DSM-24716 | Food | |
| *Lactobacillus kisonensis* | DSM-19906 | Food | |
| *Lactobacillus koreensis* | JCM-16448 | Food | |
| *Lactobacillus mindensis* | DSM-14500 | Food | |

**Supplementary Table 1.** Continued

| | | | |
|---|---|---|---|
| *Lactobacillus namurensis* | DSM-19117 | Food | |
| *Lactobacillus nantensis* | DSM-16982 | Food | Yes |
| *Lactobacillus nodensis* | DSM-19682 | Food | Yes |
| *Lactobacillus odoratitofui* | DSM-19909 | Food | |
| *Lactobacillus otakiensis* | DSM-19908 | Food | |
| *Lactobacillus panis* | DSM-6035 | Food | |
| *Lactobacillus parabrevis* | LMG-11984 | Food | |
| *Lactobacillus parabrevis* | ATCC-53295 | Food | |
| *Lactobacillus paracasei tolerans* | DSM-20258 | Food | |
| *Lactobacillus parakefiri* | DSM-10551 | Food | |
| *Lactobacillus paralimentarius* | DSM-13238 | Food | |
| *Lactobacillus paralimentarius* | DSM-13961 | Food | Yes |
| *Lactobacillus perolens* | DSM-12744 | Food | |
| *Lactobacillus plantarum plantarum* | CGMCC-1.2437 | Food | Yes |
| *Lactobacillus pontis* | DSM-8475 | Food | |
| *Lactobacillus rapi* | DSM-19907 | Food | |
| *Lactobacillus rossiae* | DSM-15814 | Food | |
| *Lactobacillus sakei carnosus* | DSM-15831 | Food | |
| *Lactobacillus sanfranciscensis* | DSM-20451 | Food | |
| *Lactobacillus secaliphilus* | DSM-17896 | Food | |
| *Lactobacillus selangorensis* | DSM-13344 | Food | |
| *Lactobacillus selangorensis* | ATCC-BAA-66 | Food | |
| *Lactobacillus senmaizukei* | DSM-21775 | Food | |
| *Lactobacillus siliginis* | DSM-22696 | Food | |
| *Lactobacillus spicheri* | DSM-15429 | Food | |
| *Lactobacillus suebicus* | DSM-5007 | Food | |
| *Lactobacillus sunkii* | DSM-19904 | Food | |
| *Lactobacillus tucceti* | DSM-20183 | Food | |
| *Lactobacillus versmoldensis* | DSM-14857 | Food | |
| *Lactobacillus xiangfangensis* | LMG-26013 | Food | |
| *Lactobacillus zymae* | DSM-19395 | Food | |
| *Lactococcus lactis* | LMG-7760 | Food | |
| *Leuconostoc argentinum* | KCTC-3773 | Food | Yes |
| *Leuconostoc carnosum* | JB16 | Food | |
| *Leuconostoc citreum* | KM20 | Food | |
| *Leuconostoc fallax* | KCTC-3537 | Food | Yes |
| *Leuconostoc gasicomitatum* | LMG-18811 | Food | |
| *Leuconostoc gelidum* | KCTC-3527 | Food | Yes |
| *Leuconostoc kimchii* | IMSNU-11154 | Food | |
| *Leuconostoc mesenteroides* | ATCC-8293 | Food | |
| *Leuconostoc pseudomesenteroides* | 4882 | Food | |
| *Pediococcus argentinicus* | DSM-23026 | Food | |
| *Weissella halotolerans* | DSM-20190 | Food | |
| *Weissella minor* | DSM-20014 | Food | |
| *Weissella viridescens* | DSM-20410 | Food | |

**Supplementary Table 1.** Continued

| | | | |
|---|---|---|---|
| *Atopobium minutum* | DSM-20586 | Human/Animal | |
| *Atopobium rimae* | DSM-7090 | Human/Animal | |
| *Carnobacterium maltaromaticum* | DSM-20730 | Human/Animal | Yes |
| *Kandleria vitulina* | DSM-20405 | Human/Animal | Yes |
| *Lactobacillus acidophilus* | DSM-20079 | Human/Animal | Yes |
| *Lactobacillus amylophilus* | DSM-20533 | Human/Animal | Yes |
| *Lactobacillus amylotrophicus* | DSM-20534 | Human/Animal | |
| *Lactobacillus amylovorus* | DSM-20531 | Human/Animal | Yes |
| *Lactobacillus amylovorus* | DSM-16698 | Human/Animal | Yes |
| *Lactobacillus animalis* | DSM-20602 | Human/Animal | |
| *Lactobacillus antri* | DSM-16041 | Human/Animal | |
| *Lactobacillus apodemi* | DSM-16634 | Human/Animal | Yes |
| *Lactobacillus aviarius araffinosus* | DSM-20653 | Human/Animal | |
| *Lactobacillus aviarius aviarius* | DSM-20655 | Human/Animal | |
| *Lactobacillus brantae* | DSM-23927 | Human/Animal | |
| *Lactobacillus brevis* | DSM-20054 | Human/Animal | |
| *Lactobacillus ceti* | DSM-22408 | Human/Animal | |
| *Lactobacillus coleohominis* | DSM-14060 | Human/Animal | |
| *Lactobacillus crispatus* | DSM-20584 | Human/Animal | Yes |
| *Lactobacillus equi* | DSM-15833 | Human/Animal | |
| *Lactobacillus equicursoris* | DSM-19284 | Human/Animal | Yes |
| *Lactobacillus equigenerosi* | DSM-18793 | Human/Animal | |
| *Lactobacillus fermentum* | DSM-20055 | Human/Animal | |
| *Lactobacillus gallinarum* | DSM-10532 | Human/Animal | Yes |
| *Lactobacillus gasseri* | ATCC-33323 | Human/Animal | |
| *Lactobacillus gastricus* | DSM-16045 | Human/Animal | Yes |
| *Lactobacillus gigeriorum* | DSM-23908 | Human/Animal | Yes |
| *Lactobacillus hamsteri* | DSM-5661 | Human/Animal | Yes |
| *Lactobacillus hayakitensis* | DSM-18933 | Human/Animal | |
| *Lactobacillus hominis* | DSM-23910 | Human/Animal | Yes |
| *Lactobacillus iners* | DSM-13335 | Human/Animal | |
| *Lactobacillus ingluviei* | DSM-15946 | Human/Animal | |
| *Lactobacillus ingluviei* | DSM-14792 | Human/Animal | |
| *Lactobacillus intestinalis* | DSM-6629 | Human/Animal | Yes |
| *Lactobacillus jensenii* | DSM-20557 | Human/Animal | |
| *Lactobacillus johnsonii* | ATCC-33200 | Human/Animal | Yes |
| *Lactobacillus kalixensis* | DSM-16043 | Human/Animal | Yes |
| *Lactobacillus kitasatonis* | DSM-16761 | Human/Animal | Yes |
| *Lactobacillus mucosae* | DSM-13345 | Human/Animal | |
| *Lactobacillus murinus* | DSM-20452 | Human/Animal | Yes |
| *Lactobacillus oligofermentans* | DSM-15707 | Human/Animal | |
| *Lactobacillus oris* | DSM-4864 | Human/Animal | |
| *Lactobacillus pantheris* | DSM-15945 | Human/Animal | |
| *Lactobacillus parabuchneri* | DSM-5707 | Human/Animal | Yes |
| *Lactobacillus psittaci* | DSM-15354 | Human/Animal | |
| *Lactobacillus rennini* | DSM-20253 | Human/Animal | |

**Supplementary Table 1.** Continued

| | | | |
|---|---|---|---|
| *Lactobacillus reuteri* | DSM-20016 | Human/Animal | |
| *Lactobacillus ruminis* | DSM-20403 | Human/Animal | |
| *Lactobacillus saerimneri* | DSM-16049 | Human/Animal | |
| *Lactobacillus salivarius* | DSM-20555 | Human/Animal | |
| *Lactobacillus saniviri* | DSM-24301 | Human/Animal | |
| *Lactobacillus senioris* | DSM-24302 | Human/Animal | |
| *Lactobacillus ultunensis* | DSM-16047 | Human/Animal | Yes |
| *Lactobacillus vaccinostercus* | DSM-20634 | Human/Animal | |
| *Lactobacillus vaginalis* | DSM-5837 | Human/Animal | |
| *Olsenella uli* | DSM-7084 | Human/Animal | |
| *Fructobacillus fructosus* | DSM-20349 | Plant | |
| *Lactobacillus buchneri* | DSM-20057 | Plant | |
| *Lactobacillus cacaonum* | DSM-21116 | Plant | |
| *Lactobacillus camelliae* | DSM-22697 | Plant | |
| *Lactobacillus coryniformis coryniformis* | DSM-20001 | Plant | |
| *Lactobacillus dextrinicus* | DSM-20335 | Plant | |
| *Lactobacillus diolivorans* | DSM-14421 | Plant | |
| *Lactobacillus fabifermentans* | DSM-21115 | Plant | |
| *Lactobacillus floricola* | DSM-23037 | Plant | |
| *Lactobacillus florum* | DSM-22689 | Plant | |
| *Lactobacillus ghanensis* | DSM-18630 | Plant | |
| *Lactobacillus graminis* | DSM-20719 | Plant | Yes |
| *Lactobacillus hokkaidonensis* | DSM-26202 | Plant | Yes |
| *Lactobacillus hordei* | DSM-19519 | Plant | Yes |
| *Lactobacillus manihotivorans* | DSM-13343 | Plant | |
| *Lactobacillus nasuensis* | JCM-17158 | Plant | |
| *Lactobacillus ozensis* | DSM-23829 | Plant | |
| *Lactobacillus plantarum* | DSM-13273 | Plant | Yes |
| *Lactobacillus plantarum argentoratensis* | DSM-16365 | Plant | |
| *Lactobacillus pobuzihii* | NBRC-103219 | Plant | |
| *Lactobacillus pobuzihii.Chen* | KCTC-13174 | Plant | |
| *Lactobacillus sucicola* | DSM-21376 | Plant | |
| *Lactobacillus taiwanensis* | DSM-21401 | Plant | Yes |
| *Lactobacillus thailandensis* | DSM-22698 | Plant | |
| *Lactobacillus uvarum* | DSM-19971 | Plant | |
| *Lactobacillus vini* | DSM-20605 | Plant | |
| *Pediococcus lolii* | DSM-19927 | Plant | |
| *Pediococcus parvulus* | DSM-20332 | Plant | |
| *Pediococcus stilesii* | DSM-18001 | Plant | |
| *Weissella confusa* | DSM-20196 | Plant | |
| *Lactobacillus fructivorans* | DSM-20203 | Unknown | |
| *Lactobacillus paracasei paracasei* | DSM-5622 | Unknown | Yes |
| *Lactobacillus pasteurii* | DSM-23907 | Unknown | Yes |
| *Lactobacillus pentosus* | DSM-20314 | Unknown | Yes |
| *Lactobacillus rhamnosus* | DSM-20021 | Unknown | Yes |
| *Leuconostoc mesenteroides cremoris* | ATCC-19254 | Unknown | |

**Supplementary Table 1.** Continued

| | | | |
|---|---|---|---|
| *Pediococcus acidilactici* | AS1-2696 | Unknown | |
| *Lactobacillus amylolyticus* | DSM-11664 | Wine/Alcohol Products | Yes |
| *Lactobacillus composti* | DSM-18527 | Wine/Alcohol Products | |
| *Lactobacillus delbrueckii jakobsenii* | DSM-26046 | Wine/Alcohol Products | |
| *Lactobacillus farraginis* | DSM-18382 | Wine/Alcohol Products | Yes |
| *Lactobacillus fructivorans* | ATCC-27394 | Wine/Alcohol Products | |
| *Lactobacillus fructivorans* | DSM-20350 | Wine/Alcohol Products | |
| *Lactobacillus helveticus* | LMG-22464 | Wine/Alcohol Products | Yes |
| *Lactobacillus hilgardii* | DSM-20176 | Wine/Alcohol Products | |
| *Lactobacillus homohiochii* | DSM-20571 | Wine/Alcohol Products | |
| *Lactobacillus kunkeei* | DSM-12361 | Wine/Alcohol Products | |
| *Lactobacillus lindneri* | DSM-20690 | Wine/Alcohol Products | |
| *Lactobacillus malefermentans* | DSM-5705 | Wine/Alcohol Products | |
| *Lactobacillus mali* | DSM-20444 | Wine/Alcohol Products | |
| *Lactobacillus mali* | ATCC-27304 | Wine/Alcohol Products | |
| *Lactobacillus nagelii* | DSM-13675 | Wine/Alcohol Products | |
| *Lactobacillus oeni* | DSM-19972 | Wine/Alcohol Products | |
| *Lactobacillus parabuchneri* | DSM-15352 | Wine/Alcohol Products | |
| *Lactobacillus parafarraginis* | DSM-18390 | Wine/Alcohol Products | Yes |
| *Lactobacillus paralimentarius* | DSM-19674 | Wine/Alcohol Products | |
| *Lactobacillus paraplantarum* | DSM-10667 | Wine/Alcohol Products | Yes |
| *Lactobacillus paucivorans* | DSM-22467 | Wine/Alcohol Products | |
| *Lactobacillus sakei sakei* | DSM-20017 | Wine/Alcohol Products | |
| *Lactobacillus satsumensis* | DSM-16230 | Wine/Alcohol Products | |
| *Lactobacillus similis* | DSM-23365 | Wine/Alcohol Products | |
| *Lactobacillus zeae* | DSM-20178 | Wine/Alcohol Products | Yes |
| *Oenococcus kitaharae* | DSM-17330 | Wine/Alcohol Products | |
| *Oenococcus oeni* | ATCC-BAA-1163 | Wine/Alcohol Products | Yes |
| *Pediococcus cellicola* | DSM-17757 | Wine/Alcohol Products | |
| *Pediococcus claussenii* | DSM-14800 | Wine/Alcohol Products | Yes |
| *Pediococcus damnosus* | DSM-20331 | Wine/Alcohol Products | Yes |
| *Pediococcus ethanolidurans* | DSM-22301 | Wine/Alcohol Products | |
| *Pediococcus inopinatus* | DSM-20285 | Wine/Alcohol Products | |
| *Pediococcus pentosaceus* | DSM-20336 | Wine/Alcohol Products | |

**Supplementary Table 2** Bacteriocin Genes Including Leader Sequences

| Species | Strain | Potential Unmodified Lantibiotic Peptide |
|---|---|---|
| Lantibipeptides | | |
| *L. taiwanensis* | DSM 21401 | MKELNLDVEKFDLADNLEVILEANGV-TSTGCCNGPSKLQG |
| *L. amylovorus* | DSM 20531 | MTESALLDLKIDESAVNAFDAEGY-AKSYSAYSSCSCVNPPCPIATMD |
| *L. gastricus* | DSM 16045 | MSKEELNSIVGRAFEEMDIQQMTSIQGS-GTETAQSTPAISRVTLSIARKSSAKCISWISFSAGGLNSYKSKC |
| *P. damnosus* (Pediocin PD-1) | DSM 20331 | MKKNLMKSAEESSGNVLEELNNAQLGMISGG-KKIKKSSSGDICTLTSECDHLATWVCC |
| Class IIa Bacteriocins | | |
| *L. hordei* | DSM 19519 | MKKIEKLTEKEMANIIGG-KYYGNGVTCGKHSCSVDWGKATTCIINNGAMAWATGGHQGTHKC |
| | | MKKEIELSEKELVRIIGG-KYYGNGVSCTKKHGCKVNWGQAFTCSVNRFANFGHGNC |
| *L. acidipiscis* | DSM 15836 | LSLEESSSVIGG-KYYGNGLHIPKHGKPYINWGQAIQSIGKISYHGWVNGITSGAAGVGRH |
| *L. futsaii* | JCM 17355 | MKGRYVNMKKVIDENSLSLISGG-KYYGNGVSCGKHTCKVNWGQAWNESVNRWGNSWVNGLTGLRQH |
| *C. maltaromaticum* | DSM 20722 | MKSVKELNKKEMQQIN-AISYGNGVYCNKEKCWVNKAENKQAITGIVIGGWASSLAGMGH |
| | | MNSVKELNVKEMKQLHGG-VYYGNGVSCSKTKCSVNWGQAFQERYTAGINSFVSGVASGAGSIGRRP |
| *L. agilis* | DSM 20509 | MSDKMENKKKLTTADLAKVTGG-SRYYGNGITCGKHKCTVNWGQAWTCGVNRLANFGHGNC |
| *L. aquaticus* | DSM 21051 | MNGG-KNYGNGVYCTKKHGYKVDWGQAWSIIGNNSAANSTTRGAAGWKSK |
| *L. rennini* | DSM 20253 | MLSKEELTQVNGG-KYYGNGVSCSKHSCSVDWGKALTCTINNGAMAWTTGGHQGNHKC |
| *L. ruminis* | DSM 20403 | MRQLSEKELKKIMGG-KYYGNGVYCGKHKCRVDWGQAWGCSVNRWGAAVGTGGKATIGHC |
| *P. pentosaceus* | DSM 20336 | MKKIEKLTEKEMANIIGG-KYYGNGLYCGKHSCSVDWGKATTCIINNGAMAWATGGHQGTHKC |
| *C. maltaromaticum* | DSM 20342 | MKSVKELNKKEMQQINGG-AISYGNGVYCNKEKCWVNKAENKQAITGIVIGGWASSLAGMGH |
| Class IIb Bacteriocins | | |
| *L. murinus* | DSM 20452 | MENLQMFDVMTEEELTEINGG-YNRLAGQIGHYTGKAVIVGATVLGIASLF |
| | | MNNAELNLFEELSTSDLQNIVGG-KRGLGYHIVDAVVSFGKGFLDAF |
| | | MNKFQVITDLSQIKGG-YDIEKALWGGYGYQLGWRNKWNLSHRYFKI |
| | | MEKLEDFKPLDTKATSSIVGG-GVPGWYYGMLWKIGVSGYKHRKDIMNGFDRGFNNYPK |
| *L. acidophilus* | DSM 20079 | MNKFKDLNELELSNIAGG-SNNIFWTRVGVGWAAEARCMIKPSLGNWTTKAVSCGAKGLYAAVRG |
| | | MKKKILESDLKNIKGG-VAPIVYPIAGYVMKQMFEHSDQIIKGFKRGWKKYK |
| *L. taiwanensis* | DSM 21401 | MKLNDKELSKIVGG-NRWGDTVLSAASGAGTGIKACKSFGPWGMAICGSNRRLFWLYS |
| | | MKQFNYLSHKDLAVVVGG-RNNWQTNVGGAVGSAMIGATVGGTICGPACAVAGAHYLPILWTGVTAATGGFGKIRK |
| *L. crispatus* | DSM 20584 | MKQLNSEQLQNIIGG-NRWTNAYSAALGCAVPGVKYGKKLGGVWGAVIGGVGGAAVCGLAGYVRKG |
| | | MSKVEVLNEEELTTVVGG-SKGKGRNNWAGNTIGIVSSAATGAALGSAICGPGCGFVGAHWGAVGWTAVASFSGAFGKIRK |
| *L. nantensis* | DSM 16982 | MKSLNKFSTISEKNLDNCNGG-SFKGFVQGFINGLTGKKH |
| | | MKKYKKIDNNTLKNISGG-KGPWNYKTGYNLGKWISKRF |
| *L. apodemi* | DSM 16634 | MNKFQVITDLNQIKGG-YDIEKALWKGYGYQLGWRSKWNLSHRYFKI |
| | | MDKLESFKLLDTKATSSIVGG-GVPGWYYSMLWKIGVSGYKHRKDIMSGFDKGFNNYPK |
| *L. plantarum* | DSM 13273 | MKIKLTVLNEFEELTADAEKNISGG-RRSRKNGIGYAIGYAFGAVERAVLGGSRDYNK |
| | | MTVNKMIKDLDVVDAFAPISNNKLNGVVGG-GAWKNFWSSLRKGFYDGEAGRAIRR |
| | | MLQFEKLQYSRLPQKKLAKISGG-FNRGGYNFGKSVRHVVDAIGSVAGIRGILKSIR |
| | | MKKFLVLRDRELNAISGG-VFHAYSARGVRNNYKSAVGPADWVISAVRGFIHG |
| *L. plantarum* subsp. *plantarum* | CGMCC 1.2437 | MKIKLTVLNEFEELTADAEKNISGG-RRSRKNGIGYAIGYAFGAVERAVLGGSRDYNK |
| | | MTVNKMIKDLDVVDAFAPISNNKLNGVVGG-GAWKNFWSSLRKGFYDGEAGRAIRR |
| | | MLQFEKLQYSRLPQKKLAKISGG-FNRGGYNFGKSVRHVVDAIGSVAGIRGILKSIR |
| | | MKKFLVLRDRELNAISGG-VFHAYSARGVRNNYKSAVGPADWVISAVRGFIHG |
| *L. paraplantarum* | DSM 10667 | LPQKKLAKISGG-FNRGGYNFGKSVRHVVDAIGSVAGIRGILKSIR |
| | | MKKFLVLRGRELNAISGG-VFHAYSARGVRNNYKSAVGPADWVISAVRGFIHG |
| *L. intestinalis* | DSM 6629 | MVTKFKTMNLKELENTIGG-RHSVPYSYGYQSGRGFKGAAAAYNIIKTVASFFE |
| | | MKDTVKFEKLDCNQLSKIVGG-KRKKHHPWYWSIQEFGRGFLAGLASKYNL |
| *L. rhamnosus* | DSM 20021 | MTKLNEAELSKISGG-IGPLAIPVAAILGFLATDAWSHADELVAGVKQGWERS |
| | | MDHLKTLNTADLASISGG-DNGNLWTFIGKAIGSTARSWAEGAMFAPAIGPAKEIVDKLNGN |
| *L. zeae* | DSM 20178 | MNTVSNRFNTVTEDELSVVSGG-NAWGNAVNGALNGAATGARFGKNLGPWGMIGGMALGAGIGGYFGYNG |
| | | MDSELTDVVGG-RNTWQQNVSGVAGAAAGGAALGAVVGGPAGAFLGAHYGPILWTAVTGFTGGF |
| *Leuc. fallax* | KCTC 3537 | MEKLSEQELAKASGG-CPLLPIVVTVAASGAHFVAKDGWNHLDQIRSGWRKSGNSKW |
| | | MDFKTQPNVLSLEKLMMISGG-STDGSWEDFGAGLHKTVNTVIYAGTTVARAHTRSHQRCFTGNKW |

**Supplementary Table 2.** Continued

| Species | Strain | Potential Unmodified Lantibiotic Peptide |
|---|---|---|
| Class IId Bacteriocins | | |
| *L. paralimentarius* | DSM 13961 | MSKFKELSITELYKTSGG-NFFGGSNGYSWRDKKGHWHYTVTSGVSSTVAQIIGNGWGSAGAPGVGQR |
| *L. pentosus* | DSM 20314 | MQKIAGG-KSNTYSLQMGSVVRTATKIFKKMEW |
| *L. hokkaidonensis* | DSM 26202 | MKFKKLSEKELQKINGG-VTLSVATHSKNGLKKFFKWVRKL |
| *L. xiangfangensis* | LMG 26013 | MNSQKVLISSELSNVIGG-KLVKLYTAEPYTFYRDTRTKKIVMRQTTGYSAHLQHVIADGWVRSAHL |
| *L. paracasei* | DSM 5622 | MKQFDEQKMVNMSDEELLGFIGG-DSIRDVSPTFNKIRRWFDGLFK |
| *L. murinus* | DSM 20452 | MNKFQVITDLSQIKGG-YDIEKALWGGYGYQLGWRNKWNLSHRYFKI |
| *Leuc. kimchii* | IMSNU 11154 | MQDVKYRELSEAELSVIAGG-KSFWSWASDASSWLSGPQQPNSPLLKKKR |
| *Leuc. geldium* | KCTC 3527 | MKSLQDFQTMNHTQLAQVNGG-KRVYIPNGNGAWLDSNTGKGGVDWNVAVPALGSIMVNGWAQNGPLAHLHP |
| *C. maltaromaticum* (cbnX) | DSM 20722 | MKSVKELNVKEMQQTIGG-WGWKEVVQNGQTIFSAGQKLGNMVGKIVPLPFG |
| Lactococcin 972 like Bacteriocins | | |
| *L. equicursoris* | DSM 19284 | MNKIKSFAVAVFATTAIAFSAVPVLASSYVIDAQ-GGTWNYGVGSKYVWSYYSHNSKTHKASVEGKYYVTSGWIKEKTQARASAAKAAAGNQSYYDVK |
| *L. amylophilus* | DSM 20533 | MKSAKKLMTAFAVTAGLLGTGVGSAVMAASVG-GGTWNYGVGLTGTFGYSDYLHNSKTHSASVGRTKSDCNKVTKTKGVWAQSKYTKIPPTGLNYWWSVS |
| *L. graminis* | DSM 20719 | MASAQAASADIPIASGQMELTQGQTPIMSCISKG-GGTWYSGFSGTKVYSQYYHGSKKHSATAKNGWGAGVRNTQKAGIWAYSSVNSTLTGNKTYWAVY |
| *L. hamsteri* | DSM 5661 | MKKSKKLIASLTATCAAVMLSTSPVLAATVYEH-GGVWNYGVGKKYVWSYYSHHRLTHKSSVEGKYYSSSGWVSPGTEARASAEKAQHGNKSYFDVE |
| *Leuc. argentinum* | KCTC 3773 | MNKFKKVVLTGVIASGIFSGISFAHADQVG-GGDWRHGVGSYYVWSYYFHNYRNHSSSVSGQYFASSGRTSPGYDAQASAPKSLFGNKAYYDFW |

**Supplementary Table 3.** Bacterial strains screened for bacteriocin production

| Species | Strain | Growth Medium | Condition | Temp (˚C) |
|---|---|---|---|---|
| *Carnobacterium maltaromaticum* | DSM 20342 | TSA | Aerobic | 30 |
| *Carnobacterium maltaromaticum* | DSM 20722 | TSA | Aerobic | 30 |
| *Carnobacterium maltaromaticum* | DSM 20730 | TSA | Aerobic | 30 |
| *Lactobacillus acidipiscis* | DSM 15836 | MRS + Vitamin soln. | Anaerobic | 30 |
| *Lactobacillus acidophilus* | DSM 20079 | mMRS | Anaerobic | 37 |
| *Lactobacillus agilis* | DSM 20509 | mMRS | Anaerobic | 38 |
| *Lactobacillus amylophilus* | DSM 20533 | MRS | Aerobic | 30 |
| *Lactobacillus amylovorus* | DSM 16698 | MRS | Anaerobic | 37 |
| *Lactobacillus amylovorus* | DSM 20531 | MRS | Anaerobic | 37 |
| *Lactobacillus apodemi* | DSM 16634 | MRS | Anaerobic | 37 |
| *Lactobacillus aquaticus* | DSM 21051 | MRS | Anaerobic | 37 |
| *Lactobacillus buchneri* | DSM 20057 | MRS | Aerobic | 37 |
| *Lactobacillus casei* | DSM 20011 | MRS | Aerobic | 30 |
| *Lactobacillus composti* | DSM 18527 | MRS | Anaerobic | 30 |
| *Lactobacillus coryniformis* subsp. *coryniformis* | DSM 20001 | MRS | Aerobic | 30 |
| *Lactobacillus coryniformis* subsp. *torquens* | DSM 20004 | MRS | Aerobic | 30 |
| *Lactobacillus crispatus* | DSM 20584 | mMRS | Anaerobic | 37 |
| *Lactobacillus equicursoris* | DSM 19284 | MRS | Anaerobic | 37 |
| *Lactobacillus fabifermentans* | DSM 21115 | MRS | Anaerobic | 30 |
| *Lactobacillus fuchuensis* | DSM 14340 | MRS | Aerobic | 20 |
| *Lactobacillus futsaii* | JCM 17355 | MRS | Aerobic | 30 |
| *Lactobacillus gastricus* | DSM 16045 | MRS | Anaerobic | 37 |
| *Lactobacillus graminis* | DSM 16045 | MRS | Aerobic | 30 |
| *Lactobacillus hamsteri* | DSM 5661 | MRS | Anaerobic | 37 |
| *Lactobacillus harbinensis* | DSM 16991 | MRS | Anaerobic | 37 |
| *Lactobacillus helveticus* | CGMCC 1.1877 | MRS | Anaerobic | 37 |
| *Lactobacillus helveticus* | LMG 22464 | MRS | Anaerobic | 37 |
| *Lactobacillus hokkaidonensis* | DSM 26202 | MRS | Anaerobic | 25 |
| *Lactobacillus hordei* | DSM 19519 | MRS | Anaerobic | 30 |
| *Lactobacillus intestinalis* | DSM 6629 | MRS | Aerobic | 37 |
| *Lactobacillus johnsoni* | DSM 10533 | MRS | Anaerobic | 37 |
| *Lactobacillus kalixensis* | DSM 16043 | MRS | Aerobic | 37 |
| *Lactobacillus kimchicus* | JCM 15530 | MRS | Aerobic | 37 |
| *Lactobacillus kimchiensis* | DSM 24716 | MRS | Anaerobic | 25 |
| *Lactobacillus kitasatonis* | DSM 16761 | MRS | Anaerobic | 37 |
| *Lactobacillus mali* | DSM 20444 | MRS | Aerobic | 30 |
| *Lactobacillus mindensis* | DSM 14500 | MRS | Anaerobic | 30 |
| *Lactobacillus murinus* | DSM 20452 | MRS | Aerobic | 37 |
| *Lactobacillus nantensis* | DSM 16982 | mMRS + 1% maltose + 0.5% YE | Aerobic | 30 |
| *Lactobacillus nodensis* | DSM 19682 | MRS | Anaerobic | 30 |
| *Lactobacillus otakiensis* | DSM 19908 | MRS | Anaerobic | 30 |

**Supplementary Table 3.** Continued

| Species | Strain | Growth Medium | Condition | Temp (˚C) |
|---|---|---|---|---|
| *Lactobacillus parabuchneri* | DSM 5707 | MRS | Aerobic | 30 |
| *Lactobacillus paracasei* subsp. *paracasei* | DSM 5622 | MRS | Aerobic | 30 |
| *Lactobacillus paracasei* subsp. *tolerans* | DSM 20258 | MRS | Aerobic | 30 |
| *Lactobacillus parafarraginis* | DSM 18390 | MRS | Anaerobic | 30 |
| *Lactobacillus paralimentarius* | DSM 13961 | MRS | Aerobic | 30 |
| *Lactobacillus paralimentarius* | DSM 13238 | MRS | Aerobic | 30 |
| *Lactobacillus paraplantarum* | DSM 10667 | MRS | Aerobic | 30 |
| *Lactobacillus pasteurii* | DSM 23907 | MRS | Anaerobic | 37 |
| *Lactobacillus pentosus* | DSM 20314 | MRS | Aerobic | 30 |
| *Lactobacillus plantarum* | DSM 13273 | MRS | Aerobic | 37 |
| *Lactobacillus plantarum* subsp. *argentoratensis* | DSM 16365 | MRS | Anaerobic | 30 |
| *Lactobacillus plantarum* subsp. *plantarum* | DSM 20174 | MRS | Aerobic | 30 |
| *Lactobacillus rennini* | DSM 20253 | MRS + CAA | Aerobic | 30 |
| *Lactobacillus reuteri* | DSM 20016 | MRS | Aerobic | 37 |
| *Lactobacillus rhamnosus* | DSM 20021 | MRS | Aerobic | 37 |
| *Lactobacillus rossiae* | DSM 15814 | MRS + 1% maltose + 1% YE | Anaerobic | 30 |
| *Lactobacillus ruminis* | DSM 20403 | MRS | Anaerobic | 37 |
| *Lactobacillus similis* | DSM 23365 | MRS | Anaerobic | 37 |
| *Lactobacillus taiwanensis* | DSM 21401 | MRS | Anaerobic | 37 |
| *Lactobacillus xiangfangensis* | LMG 26013 | MRS | Aerobic | 30 |
| *Lactobacillus zeae* | DSM 20178 | MRS | Aerobic | 37 |
| *Leuconostoc fallax* | DSM 20189 | MRS | Aerobic | 30 |
| *Leuconostoc gasicomitatum* | LMG 18811 | MRS | Anaerobic | 22 |
| *Leuconostoc gelidum* subsp. *gelidum* | DSM 5578 | MRS | Anaerobic | 25 |
| *Leuconostoc lactis* | DSM 8581 | MRS | Aerobic | 30 |
| *Oenococcus kitaharae* | DSM 17330 | mMRS + 10% tomato juice | Anaerobic | 30 |
| *Oenococcus oeni* | ATCC-BAA 1163 | MRS | Anaerobic | 37 |
| *Pediococcus cellicola* | DSM 17757 | MRS | Anaerobic | 30 |
| *Pediococcus claussenii* | DSM 14800 | MRS pH 5.7 | Aerobic | 30 |
| *Pediococcus damnosus* | DSM 20331 | mMRS pH 5.7 | Anaerobic | 26 |
| *Pediococcus ethanolidurans* | DSM 22301 | MRS | Anaerobic | 37 |
| *Pediococcus pentosaceus* | DSM 20336 | MRS | Aerobic | 30 |
| *Pediococcus stilesii* | DSM 18001 | MRS | Anaerobic | 30 |

**Supplementary Table 4. Indicator Strains**

| Species | Strain | Growth Medium | Conditions | Temp (˚C) |
|---|---|---|---|---|
| *Enterococcus faecium* | LMG 11423 | MRS | Anaerobic | 37 |
| *E. mundtii* | LMG 10748 | MRS | Anaerobic | 37 |
| *E. saccharolyticus* | LMG 11427 | MRS | Anaerobic | 37 |
| *Lactobacillus acidophilus* | LMG 9433 | MRS | Anaerobic | 37 |
| *L. agilis* | LMG 9186 | MRS | Anaerobic | 37 |
| *L. amylovorus* | LMG 9496 | MRS | Anaerobic | 37 |
| *L. casei* | LMG 6904 | mMRS | Anaerobic | 37 |
| *L. crispatus* | LMG 9479 | MRS | Anaerobic | 37 |
| *L. delbrueckii* subsp. *bulgaricus* | LMG 6901 | MRS | Anaerobic | 37 |
| *L. delbrueckii* subsp. *lactis* | LMG 7942 | MRS | Anaerobic | 37 |
| *L. fermentum* | LMG 6902 | MRS | Anaerobic | 37 |
| *L. johnsonii* | DSM 10533 | MRS | Anaerobic | 37 |
| *L. plantarum* | LMG 6907 | MRS | Anaerobic | 37 |
| *L. rhamnosus* | GG | MRS | Anaerobic | 37 |
| *Listeria innocua* | DPC 3572 | BHI | Aerobic | 37 |
| *Micrococcus luteus* | DPC 6275 | BHI | Aerobic | 30 |
| *Staphylococcus aureus* | DPC 5246 | BHI | Aerobic | 37 |
| *Salmonlella typhimurium* | LT2 | BHI | Aerobic | 37 |

**Supplementary Figure 1.** Divergence and percentage identity of helveticin homologs encoded by *Lactobacillus* species (Clustal ω)

Percent Identity

Divergence

| | 1 | 2 | 3 | 4 | 5 | 6 | 7 | 8 | 9 | 10 | 11 | 12 | 13 | 14 | 15 | 16 | 17 | 18 | 19 | 20 | 21 | 22 | 23 | 24 | 25 | 26 | 27 | 28 | 29 | 30 | 31 | 32 | 33 | 34 | 35 | 36 | 37 | 38 | 39 | 40 | 41 | | |
|---|---|---|---|---|---|---|---|---|---|---|---|---|---|---|---|---|---|---|---|---|---|---|---|---|---|---|---|---|---|---|---|---|---|---|---|---|---|---|---|---|---|---|---|
| **1** | | 32.1 | 40.0 | 82.9 | 44.1 | 40.3 | 45.2 | 49.7 | 42.2 | 46.9 | 41.0 | 78.2 | 45.2 | 41.2 | 50.7 | 44.6 | 38.9 | 38.2 | 46.6 | 40.9 | 40.5 | 41.1 | 36.3 | 45.2 | 50.5 | 44.9 | 35.8 | 34.5 | 57.7 | 45.6 | 45.2 | 40.6 | 36.0 | 41.6 | 60.5 | 45.2 | 47.8 | 34.8 | 37.9 | 60.6 | 59.9 | **1** | *L. hamsteri* DSM5661 Hel1 |
| **2** | 147.6 | | 27.7 | 30.6 | 30.8 | 31.5 | 27.4 | 34.6 | 28.4 | 28.0 | 30.5 | 29.8 | 27.7 | 27.1 | 30.9 | 28.4 | 27.9 | 28.1 | 27.4 | 27.1 | 27.5 | 31.8 | 26.3 | 27.7 | 31.9 | 27.7 | 40.3 | 58.2 | 30.6 | 27.4 | 27.7 | 29.8 | 26.3 | 29.8 | 27.9 | 27.7 | 34.5 | 22.3 | 27.9 | 27.3 | 30.1 | **2** | *L. parabuchneri* DSM 5707 Hel1 |
| **3** | 111.5 | 175.9 | | 41.2 | 40.9 | 36.2 | 34.2 | 41.1 | 67.4 | 36.3 | 43.1 | 38.6 | 34.2 | 74.4 | 42.9 | 33.9 | 46.6 | 50.8 | 35.6 | 74.4 | 34.8 | 50.2 | 53.8 | 34.2 | 43.0 | 33.6 | 32.7 | 31.5 | 34.5 | 33.6 | 34.2 | 35.1 | 53.5 | 63.6 | 34.4 | 34.2 | 40.5 | 52.0 | 57.6 | 39.3 | 35.3 | **3** | *L. intestinalis* DSM6629 Hel 1 |
| **4** | 19.5 | 156.1 | 107.0 | | 45.7 | 40.5 | 42.5 | 49.5 | 40.4 | 43.5 | 42.5 | 73.6 | 42.5 | 42.1 | 52.6 | 43.5 | 38.7 | 38.4 | 43.8 | 41.8 | 40.7 | 44.3 | 39.2 | 42.5 | 49.7 | 42.2 | 34.5 | 34.4 | 58.8 | 43.1 | 42.5 | 40.8 | 38.9 | 41.8 | 59.1 | 42.5 | 46.9 | 33.8 | 37.8 | 61.3 | 58.8 | **4** | *L. intestinalis* DSM6629 Hel 2 |
| **5** | 97.0 | 155.0 | 108.2 | 92.1 | | 40.4 | 38.2 | 46.4 | 42.7 | 37.8 | 54.0 | 41.4 | 38.2 | 42.1 | 52.1 | 37.8 | 39.5 | 37.6 | 38.2 | 42.1 | 37.6 | 45.5 | 42.3 | 38.2 | 46.5 | 38.2 | 34.8 | 32.9 | 40.5 | 37.8 | 38.2 | 37.5 | 42.0 | 42.3 | 42.0 | 38.5 | 48.0 | 30.9 | 41.2 | 41.0 | 42.2 | **5** | *L. intestinalis* DSM6629 Hel 3 |
| **6** | 110.2 | 150.7 | 126.9 | 109.5 | 110.1 | | 37.4 | 39.1 | 38.2 | 38.7 | 39.9 | 37.6 | 37.4 | 38.0 | 42.4 | 38.4 | 30.8 | 34.2 | 38.7 | 38.3 | 70.4 | 36.8 | 35.6 | 37.4 | 39.8 | 37.0 | 33.3 | 32.3 | 33.2 | 38.0 | 37.4 | 70.1 | 35.6 | 39.4 | 34.7 | 37.4 | 39.6 | 37.3 | 36.6 | 37.8 | 36.1 | **6** | *L. intestinalis* DSM6629 Hel 4 |
| **7** | 93.5 | 178.3 | 136.3 | 102.5 | 118.6 | 122.0 | | 41.7 | 33.8 | 85.8 | 38.0 | 45.1 | 99.7 | 34.5 | 42.1 | 87.7 | 30.6 | 31.6 | 87.1 | 34.1 | 40.6 | 35.2 | 31.4 | 99.7 | 39.9 | 97.8 | 30.3 | 29.1 | 41.3 | 89.2 | 99.7 | 39.5 | 31.4 | 35.3 | 46.1 | 99.4 | 42.1 | 28.2 | 31.5 | 44.3 | 44.4 | **7** | *L. gallinarum* DSM10532 Hel 1 |
| **8** | 80.8 | 134.4 | 107.4 | 81.2 | 90.0 | 115.1 | 105.3 | | 42.5 | 43.1 | 45.0 | 46.9 | 41.7 | 43.0 | 51.9 | 42.4 | 40.6 | 38.6 | 43.1 | 42.7 | 37.2 | 45.6 | 41.9 | 41.7 | 67.0 | 42.4 | 37.2 | 34.5 | 42.2 | 42.0 | 41.7 | 38.9 | 41.5 | 41.5 | 46.8 | 41.7 | 68.9 | 33.1 | 39.4 | 46.6 | 46.8 | **8** | *L. gallinarum* DSM10532 Hel 2 |
| **9** | 103.4 | 170.5 | 42.7 | 110.0 | 101.9 | 118.6 | 138.6 | 102.4 | | 36.5 | 42.3 | 39.2 | 34.1 | 72.4 | 44.1 | 34.1 | 48.1 | 50.0 | 35.5 | 72.7 | 37.7 | 51.0 | 55.1 | 34.1 | 43.0 | 35.1 | 33.0 | 29.1 | 38.4 | 36.5 | 34.1 | 38.7 | 54.8 | 77.1 | 37.2 | 34.1 | 41.4 | 85.0 | 61.0 | 39.5 | 35.7 | **9** | *L. gallinarum* DSM10532 Hel 3 |
| **10** | 88.6 | 173.2 | 126.8 | 99.2 | 120.0 | 116.4 | 15.7 | 100.4 | 125.8 | | 38.6 | 46.4 | 86.2 | 36.1 | 43.2 | 83.4 | 31.9 | 33.6 | 88.9 | 35.8 | 43.2 | 36.2 | 33.4 | 86.2 | 39.6 | 86.2 | 32.1 | 30.7 | 42.0 | 85.8 | 86.2 | 41.9 | 33.4 | 37.6 | 46.7 | 85.8 | 41.7 | 31.0 | 33.9 | 44.9 | 45.4 | **10** | *L. kefiranofaciens kefirgranum* DSM10550 Hel 1 |
| **11** | 107.8 | 156.8 | 100.5 | 102.4 | 69.9 | 112.0 | 119.5 | 94.1 | 103.0 | 116.7 | | 39.3 | 38.0 | 44.6 | 53.3 | 38.6 | 41.4 | 36.1 | 40.3 | 44.6 | 37.1 | 42.8 | 40.1 | 38.0 | 45.0 | 39.0 | 35.7 | 30.9 | 38.8 | 38.3 | 38.0 | 35.4 | 39.8 | 42.3 | 39.3 | 38.0 | 45.1 | 34.0 | 40.3 | 40.5 | 40.3 | **11** | *L. amylolyticus* DSM11664 Hel 1 |
| **12** | 25.8 | 161.3 | 116.7 | 32.5 | 106.4 | 120.9 | 94.1 | 88.6 | 114.6 | 90.1 | 114.0 | | 45.1 | 38.9 | 50.3 | 45.7 | 35.9 | 36.6 | 45.7 | 38.9 | 38.2 | 40.1 | 36.4 | 45.1 | 46.7 | 44.7 | 32.5 | 31.8 | 56.8 | 45.1 | 45.1 | 37.6 | 36.1 | 41.2 | 57.6 | 45.1 | 45.7 | 34.1 | 35.5 | 60.8 | 55.1 | **12** | *L. amylolyticus* DSM11664 Hel 2 |
| **13** | 93.5 | 175.7 | 136.3 | 102.5 | 118.6 | 122.0 | 0.3 | 105.3 | 136.9 | 15.3 | 119.5 | 94.1 | | 34.5 | 42.1 | 88.0 | 30.6 | 31.9 | 87.4 | 34.1 | 41.0 | 35.2 | 31.4 | 100.0 | 39.9 | 98.2 | 30.3 | 29.1 | 41.3 | 89.5 | 100.0 | 39.9 | 31.4 | 35.3 | 46.1 | 99.7 | 42.4 | 28.9 | 31.5 | 44.3 | 44.4 | **13** | *L. helveticus* CGMCC1.1877 Hel 1 |
| **14** | 107.0 | 180.0 | 31.4 | 103.9 | 103.8 | 119.4 | 135.2 | 100.8 | 34.4 | 127.3 | 95.6 | 115.9 | 135.2 | | 46.2 | 34.8 | 46.0 | 49.8 | 36.1 | 99.4 | 35.9 | 52.0 | 57.3 | 34.5 | 42.1 | 33.8 | 33.8 | 32.3 | 36.4 | 35.8 | 34.5 | 37.1 | 56.6 | 72.7 | 36.7 | 34.5 | 40.6 | 57.2 | 61.7 | 39.2 | 36.8 | **14** | *L. helveticus* CGMCC1.1877 Hel 2 |
| **15** | 78.0 | 154.7 | 101.1 | 73.1 | 74.4 | 102.9 | 103.8 | 75.0 | 97.0 | 100.3 | 71.4 | 78.9 | 103.8 | 90.6 | | 43.2 | 40.6 | 41.0 | 43.5 | 46.2 | 43.1 | 48.8 | 43.6 | 42.1 | 48.3 | 42.5 | 36.3 | 31.9 | 44.6 | 42.8 | 42.1 | 41.1 | 43.3 | 43.8 | 48.3 | 42.1 | 49.8 | 36.3 | 43.1 | 49.1 | 46.2 | **15** | *L. helveticus* CGMCC1.1877 Hel 3 |
| **16** | 95.6 | 170.8 | 138.0 | 99.2 | 120.0 | 117.8 | 13.5 | 102.8 | 136.9 | 18.8 | 116.7 | 92.1 | 13.1 | 133.6 | 100.3 | | 31.6 | 32.9 | 84.6 | 34.5 | 42.5 | 36.2 | 32.4 | 88.0 | 39.6 | 88.0 | 30.7 | 28.0 | 42.6 | 87.7 | 88.0 | 41.2 | 32.4 | 34.9 | 46.4 | 87.7 | 42.1 | 31.0 | 33.9 | 44.3 | 44.8 | **16** | *L. kalixensis* DSM16043 Hel 1 |
| **17** | 115.8 | 174.1 | 89.4 | 116.4 | 113.6 | 154.9 | 156.1 | 109.1 | 85.2 | 148.5 | 106.2 | 128.5 | 156.1 | 91.3 | 109.2 | 150.3 | | 40.9 | 31.3 | 46.3 | 32.4 | 44.8 | 40.9 | 30.6 | 41.2 | 30.9 | 31.2 | 29.0 | 36.8 | 33.2 | 30.6 | 33.4 | 40.6 | 46.0 | 35.6 | 30.6 | 41.7 | 40.7 | 43.4 | 35.9 | 35.4 | **17** | *L. kalixensis* DSM16043 Hel 2 |
| **18** | 118.6 | 172.6 | 77.7 | 117.8 | 120.9 | 136.7 | 150.5 | 116.8 | 79.9 | 139.7 | 127.7 | 125.3 | 148.6 | 80.3 | 107.9 | 143.2 | 108.2 | | 33.9 | 49.5 | 35.2 | 44.6 | 45.0 | 31.9 | 37.5 | 31.9 | 30.0 | 29.6 | 35.8 | 33.9 | 31.9 | 34.8 | 44.7 | 47.1 | 35.0 | 31.9 | 36.7 | 49.3 | 47.8 | 36.5 | 35.8 | **18** | *L. kalixensis* DSM16043 Hel 3 |
| **19** | 89.5 | 178.3 | 129.8 | 98.2 | 118.6 | 116.4 | 14.2 | 100.4 | 130.4 | 12.0 | 110.2 | 92.1 | 13.9 | 127.3 | 99.1 | 17.3 | 152.2 | 138.1 | | 35.8 | 44.0 | 35.8 | 32.8 | 87.4 | 39.9 | 87.4 | 31.8 | 29.7 | 41.3 | 86.2 | 87.4 | 42.9 | 32.8 | 36.9 | 47.1 | 87.7 | 42.1 | 29.6 | 34.2 | 45.2 | 44.8 | **19** | *L. ultunensis* DSM16047 Hel 1 |
| **20** | 108.2 | 180.0 | 31.4 | 105.1 | 103.8 | 118.0 | 136.9 | 101.9 | 33.9 | 128.8 | 95.6 | 115.9 | 136.9 | 0.6 | 90.6 | 135.2 | 90.4 | 81.1 | 128.8 | | 36.3 | 51.6 | 57.0 | 34.1 | 42.1 | 33.4 | 34.2 | 32.3 | 36.4 | 35.5 | 34.1 | 37.5 | 56.3 | 72.4 | 37.0 | 34.1 | 40.6 | 56.6 | 61.7 | 38.9 | 37.2 | **20** | *L. ultunensis* DSM16047 Hel 2 |
| **21** | 109.8 | 177.0 | 133.7 | 108.9 | 121.0 | 37.6 | 109.2 | 122.5 | 120.4 | 100.0 | 123.1 | 118.7 | 107.9 | 128.3 | 100.5 | 102.5 | 145.7 | 131.7 | 97.5 | 126.5 | | 35.3 | 35.5 | 41.0 | 38.2 | 41.4 | 31.8 | 29.3 | 34.0 | 43.6 | 41.0 | 98.2 | 35.5 | 37.5 | 37.3 | 41.0 | 38.6 | 35.1 | 33.5 | 38.8 | 35.7 | **21** | *L. amylovorus* DSM16698 Hel 1 |
| **22** | 107.5 | 149.1 | 79.4 | 96.5 | 92.7 | 124.3 | 131.9 | 92.4 | 77.3 | 127.2 | 101.6 | 111.2 | 131.9 | 74.8 | 83.1 | 127.2 | 95.0 | 95.5 | 128.7 | 75.6 | 131.1 | | 49.5 | 35.2 | 45.4 | 35.2 | 32.7 | 35.7 | 42.4 | 35.2 | 35.2 | 35.6 | 49.2 | 49.0 | 39.7 | 35.5 | 43.8 | 42.5 | 51.3 | 39.9 | 39.9 | **22** | *L. amylovorus* DSM16698 Hel 2 |
| **23** | 126.6 | 186.7 | 70.2 | 114.4 | 103.1 | 129.9 | 151.5 | 104.6 | 67.1 | 140.3 | 111.0 | 126.1 | 151.5 | 62.3 | 98.8 | 145.7 | 108.0 | 94.2 | 143.9 | 63.0 | 130.5 | 81.2 | | 31.4 | 41.4 | 32.1 | 31.9 | 31.8 | 34.2 | 32.1 | 31.4 | 36.1 | 99.4 | 52.5 | 34.6 | 31.4 | 41.7 | 46.2 | 50.0 | 38.7 | 32.9 | **23** | *L. amylovorus* DSM16698 Hel 3 |
| **24** | 93.5 | 175.7 | 136.3 | 102.5 | 118.6 | 122.0 | 0.3 | 105.3 | 136.9 | 15.3 | 119.5 | 94.1 | 0.0 | 135.2 | 103.8 | 13.1 | 156.1 | 148.6 | 13.9 | 136.9 | 107.9 | 131.9 | 151.5 | | 39.9 | 98.2 | 30.3 | 29.1 | 41.3 | 89.5 | 100.0 | 39.9 | 31.4 | 35.3 | 46.1 | 99.7 | 42.4 | 28.9 | 31.5 | 44.3 | 44.4 | **24** | *L. amylovorus* DSM16698 Hel 4 |
| **25** | 78.5 | 148.4 | 100.8 | 80.8 | 89.7 | 112.3 | 111.7 | 43.4 | 100.8 | 113.1 | 94.4 | 89.0 | 111.7 | 103.7 | 84.4 | 113.1 | 107.2 | 121.4 | 111.7 | 103.7 | 118.7 | 93.2 | 106.4 | 111.7 | | 39.6 | 36.1 | 31.9 | 43.8 | 39.6 | 39.9 | 38.2 | 41.0 | 42.0 | 44.9 | 39.9 | 72.1 | 32.9 | 40.7 | 45.4 | 47.0 | **25** | *L. kitasatonis* DSM16761 Hel 1 |
| **26** | 94.5 | 175.7 | 139.7 | 103.7 | 118.6 | 123.4 | 2.2 | 102.8 | 132.0 | 15.3 | 115.4 | 95.1 | 1.9 | 138.6 | 102.6 | 13.1 | 154.1 | 148.6 | 13.9 | 140.3 | 106.5 | 131.9 | 147.6 | 1.9 | 113.1 | | 31.0 | 29.1 | 41.0 | 90.2 | 98.2 | 40.2 | 32.1 | 35.3 | 46.4 | 97.8 | 41.7 | 29.6 | 31.5 | 44.3 | 44.4 | **26** | *L. kitasatonis* DSM16761 Hel 2 |
| **27** | 129.0 | 110.4 | 144.2 | 134.8 | 133.4 | 140.9 | 157.9 | 122.6 | 142.8 | 147.3 | 129.2 | 145.4 | 157.9 | 138.3 | 126.5 | 155.7 | 152.5 | 160.2 | 149.4 | 136.4 | 149.4 | 144.2 | 148.4 | 157.9 | 127.6 | 153.5 | | 41.7 | 32.6 | 31.8 | 30.3 | 33.1 | 31.9 | 33.7 | 37.0 | 30.0 | 38.4 | 24.8 | 30.7 | 32.5 | 35.2 | **27** | *L. farraginis* DSM18382 Hel 1 |
| **28** | 135.2 | 60.3 | 151.1 | 135.7 | 143.3 | 146.6 | 166.1 | 135.0 | 166.1 | 155.4 | 154.6 | 148.9 | 166.1 | 146.4 | 148.4 | 173.2 | 166.7 | 162.6 | 161.7 | 146.4 | 164.4 | 129.5 | 149.1 | 166.1 | 148.4 | 166.1 | 105.2 | | 32.0 | 30.1 | 29.1 | 31.2 | 31.8 | 32.9 | 31.3 | 29.1 | 32.8 | 23.6 | 31.0 | 31.4 | 32.3 | **28** | *L. parafarraginis* DSM18390 Hel 1 |
| **29** | 61.4 | 156.3 | 135.2 | 59.1 | 109.4 | 141.5 | 106.6 | 103.5 | 117.8 | 104.3 | 116.1 | 63.4 | 106.6 | 126.4 | 95.6 | 102.1 | 124.6 | 128.8 | 106.6 | 126.4 | 137.6 | 102.7 | 136.3 | 106.6 | 98.0 | 107.8 | 144.8 | 148.3 | | 42.3 | 41.3 | 34.2 | 33.9 | 38.0 | 53.4 | 41.3 | 42.2 | 33.6 | 38.8 | 53.6 | 51.9 | **29** | *L. equicursoris* DSM19284 Hel 1 |
| **30** | 92.5 | 178.3 | 139.7 | 100.3 | 120.0 | 119.2 | 11.7 | 104.0 | 125.8 | 15.7 | 118.1 | 94.1 | 11.3 | 128.8 | 101.4 | 13.5 | 141.4 | 138.1 | 15.3 | 130.4 | 98.8 | 131.9 | 147.6 | 11.3 | 113.1 | 10.6 | 149.4 | 159.5 | 103.2 | | 89.5 | 41.9 | 32.1 | 36.9 | 46.1 | 89.2 | 41.7 | 31.7 | 33.9 | 44.6 | 45.4 | **30** | *L. acidophilus* DSM20079 Hel 1 |
| **31** | 93.5 | 175.7 | 136.3 | 102.5 | 118.6 | 122.0 | 0.3 | 105.3 | 136.9 | 15.3 | 119.5 | 94.1 | 0.0 | 135.2 | 103.8 | 13.1 | 156.1 | 148.6 | 13.9 | 136.9 | 107.9 | 131.9 | 151.5 | 0.0 | 111.7 | 1.9 | 157.9 | 166.1 | 106.6 | 11.3 | | 39.9 | 31.4 | 35.3 | 46.1 | 99.7 | 42.4 | 28.9 | 31.5 | 44.3 | 44.4 | **31** | *L. amylovorus* DSM20531 Hel 1 |
| **32** | 109.2 | 161.1 | 132.4 | 108.5 | 121.6 | 38.1 | 113.2 | 115.6 | 116.5 | 104.7 | 130.7 | 121.1 | 112.0 | 123.1 | 107.6 | 107.1 | 140.3 | 133.4 | 101.3 | 121.6 | 1.8 | 129.6 | 127.4 | 112.0 | 118.4 | 110.7 | 142.2 | 152.5 | 136.4 | 104.7 | 112.0 | | 36.1 | 38.8 | 36.0 | 39.9 | 38.5 | 35.1 | 35.8 | 37.1 | 34.8 | **32** | *L. amylovorus* DSM20531 Hel 2 |
| **33** | 128.2 | 186.7 | 71.0 | 115.7 | 104.4 | 129.9 | 151.5 | 105.8 | 67.9 | 140.3 | 112.2 | 127.6 | 151.5 | 63.8 | 99.9 | 145.7 | 109.3 | 95.2 | 143.9 | 64.5 | 130.5 | 82.0 | 0.6 | 151.5 | 107.6 | 147.6 | 148.4 | 149.1 | 138.0 | 147.6 | 151.5 | 127.4 | | 52.2 | 34.2 | 31.4 | 41.4 | 46.2 | 49.7 | 38.4 | 32.5 | **33** | *L. amylovorus* DSM20531 Hel 3 |
| **34** | 105.8 | 161.3 | 49.4 | 105.1 | 103.1 | 113.9 | 131.4 | 106.0 | 27.4 | 120.9 | 103.0 | 107.0 | 131.4 | 33.9 | 98.2 | 133.0 | 91.3 | 87.8 | 123.8 | 34.4 | 121.4 | 82.5 | 73.4 | 131.4 | 104.2 | 131.4 | 139.0 | 143.3 | 119.2 | 123.8 | 131.4 | 116.0 | 74.3 | | 37.7 | 35.3 | 40.5 | 62.1 | 59.4 | 41.2 | 37.5 | **34** | *L. crispatus* DSM20584 Hel 1 |
| **35** | 55.6 | 174.1 | 135.3 | 58.5 | 104.1 | 134.1 | 91.0 | 88.9 | 122.7 | 89.0 | 114.3 | 61.6 | 91.0 | 125.0 | 84.6 | 90.0 | 129.6 | 132.6 | 88.1 | 123.6 | 122.5 | 112.5 | 134.6 | 91.0 | 94.5 | 90.0 | 123.6 | 152.2 | 71.2 | 91.0 | 91.0 | 128.0 | 136.3 | 120.7 | | 46.1 | 44.6 | 34.3 | 34.3 | 54.3 | 87.6 | **35** | *L. taiwanensis* DSM21401 Hel 1 |
| **36** | 93.5 | 175.7 | 136.3 | 102.5 | 117.2 | 122.0 | 0.6 | 105.3 | 136.9 | 15.7 | 119.5 | 94.1 | 0.3 | 135.2 | 103.8 | 13.5 | 156.1 | 148.6 | 13.5 | 136.9 | 107.9 | 130.3 | 151.5 | 0.3 | 111.7 | 2.2 | 160.2 | 166.1 | 106.6 | 11.7 | 0.3 | 112.0 | 151.5 | 131.4 | 91.0 | | 42.4 | 28.9 | 31.2 | 44.3 | 44.4 | **36** | *L. helveticus* LMG22464 Hel 1 |
| **37** | 86.0 | 135.0 | 109.7 | 88.4 | 85.4 | 112.8 | 104.0 | 40.1 | 106.2 | 105.2 | 93.8 | 92.0 | 102.8 | 109.3 | 80.3 | 104.0 | 105.3 | 124.9 | 104.0 | 109.3 | 117.1 | 98.0 | 105.2 | 102.8 | 34.9 | 105.2 | 117.6 | 143.9 | 103.6 | 105.2 | 102.8 | 117.5 | 106.4 | 109.7 | 95.5 | 102.8 | | 33.8 | 40.2 | 44.8 | 46.3 | **37** | *L. pasteurii* DSM23907 Hel 1 |
| **38** | 133.7 | 219.0 | 74.7 | 138.4 | 154.2 | 122.2 | 172.3 | 142.1 | 16.8 | 153.9 | 137.4 | 137.2 | 167.4 | 62.4 | 126.7 | 153.9 | 109.0 | 81.6 | 162.7 | 63.9 | 132.3 | 102.6 | 90.7 | 167.4 | 143.4 | 162.7 | 196.0 | 208.0 | 139.6 | 149.8 | 167.4 | 132.3 | 90.7 | 52.4 | 136.0 | 167.4 | 138.4 | | 50.3 | 35.5 | 31.4 | **38** | *L. pasteurii* DSM23907 Hel 2 |
| **39** | 119.8 | 174.5 | 61.5 | 120.4 | 106.9 | 125.2 | 150.7 | 113.6 | 54.6 | 138.0 | 110.2 | 130.3 | 150.7 | 53.1 | 100.4 | 138.0 | 99.6 | 86.0 | 136.3 | 53.1 | 140.2 | 76.4 | 79.9 | 150.7 | 108.9 | 150.7 | 155.7 | 153.9 | 116.2 | 138.0 | 150.7 | 129.1 | 80.7 | 57.9 | 135.8 | 152.7 | 110.7 | 79.0 | | 35.5 | 34.1 | **39** | *L. gigeriorum* DSM23908 Hel 1 |
| **40** | 55.3 | 178.8 | 114.1 | 53.9 | 107.9 | 120.0 | 96.6 | 89.5 | 113.3 | 94.5 | 109.7 | 54.9 | 96.6 | 114.6 | 82.2 | 96.6 | 128.5 | 125.8 | 93.5 | 115.9 | 116.2 | 112.0 | 116.5 | 96.6 | 93.0 | 96.6 | 145.4 | 151.5 | 70.8 | 95.6 | 96.6 | 123.0 | 117.9 | 107.0 | 69.1 | 96.6 | 95.0 | 130.2 | 130.3 | | 52.7 | **40** | *L. hominis* DSM23910 Hel 1 |
| **41** | 56.8 | 159.1 | 131.4 | 59.1 | 103.6 | 127.3 | 96.0 | 88.9 | 129.4 | 93.0 | 110.5 | 67.2 | 96.0 | 124.3 | 90.6 | 95.0 | 130.6 | 128.8 | 95.0 | 122.9 | 129.2 | 112.0 | 143.3 | 96.0 | 88.4 | 96.0 | 131.8 | 146.4 | 75.0 | 93.0 | 96.0 | 133.7 | 145.1 | 121.4 | 13.6 | 96.0 | 90.3 | 151.5 | 136.8 | 72.8 | | **41** | *L. johnsonii* ATCC33200 Hel 1 |
| | **1** | **2** | **3** | **4** | **5** | **6** | **7** | **8** | **9** | **10** | **11** | **12** | **13** | **14** | **15** | **16** | **17** | **18** | **19** | **20** | **21** | **22** | **23** | **24** | **25** | **26** | **27** | **28** | **29** | **30** | **31** | **32** | **33** | **34** | **35** | **36** | **37** | **38** | **39** | **40** | **41** | | |

**Supplementary Figure 2.** Colony MALDI TOF MS and SDS PAGE profiles of the peptides produced by the cells indicate the mass and identity of each bacteriocin produced

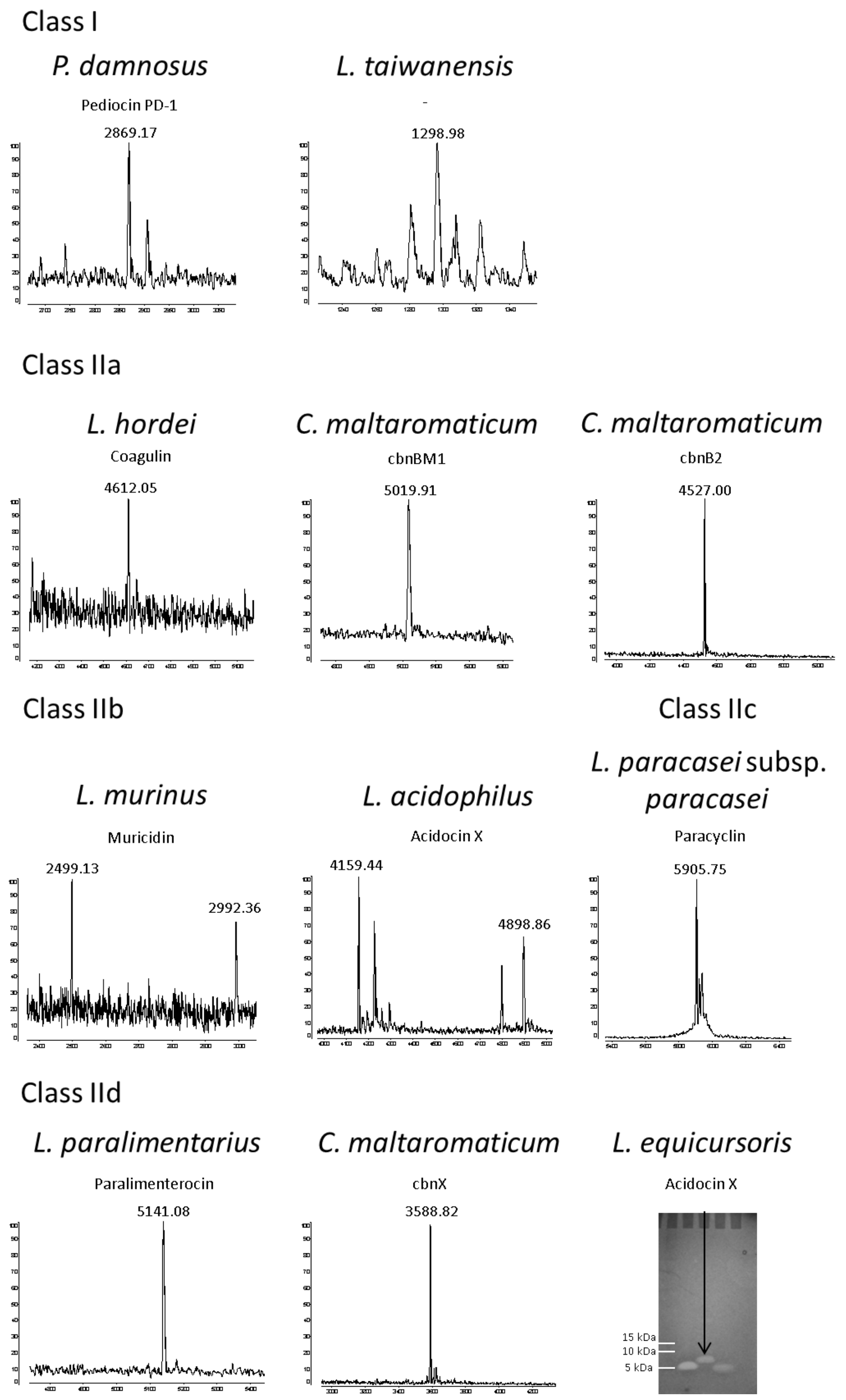

**Figure 3.** An SDS PAGE gel overlayed with the indicator *L. delbrueckii* subsp. *bulgaricus* shows the mass of the antimicrobial helveticin-like peptides produced by these lactobacilli (Unedited gel image can be seen in Supplementary Figure 4).

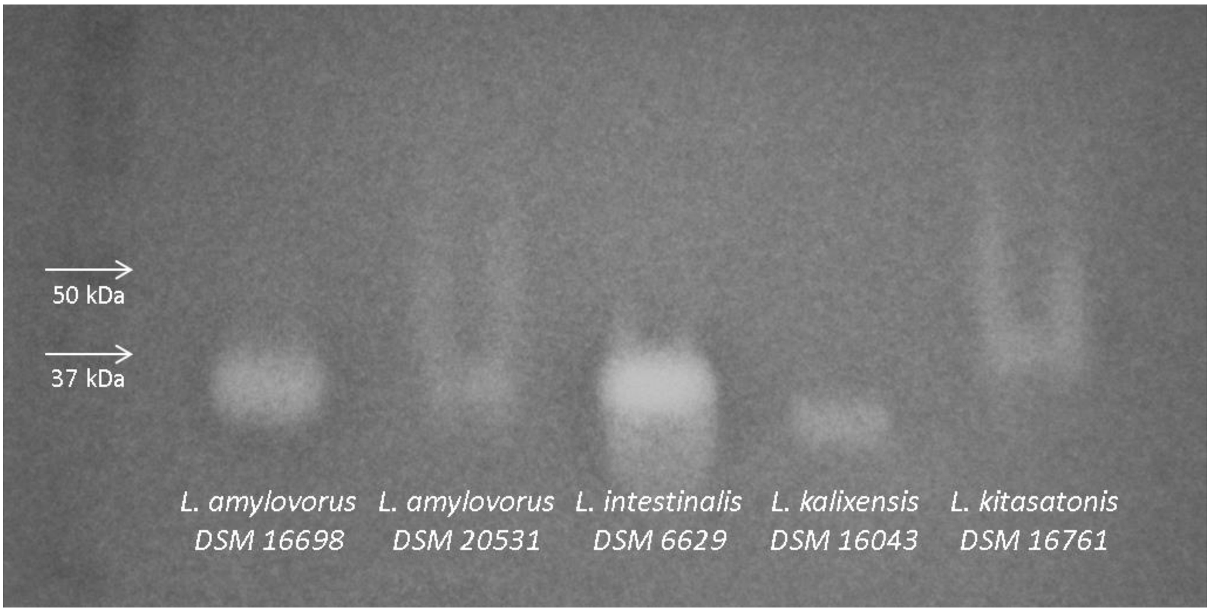

**Supplementary Figure 4.** Unedited SDS Gel overlay displaying zones of inhibition for helveticin like proteins produced by *L. amylovorus* DSM 16698, *L. amylovorus* DSM 20531, *L. intestinal* DSM 6629, *L. kalixensis* 16043 and *L. kitasatonis* DSM 16761

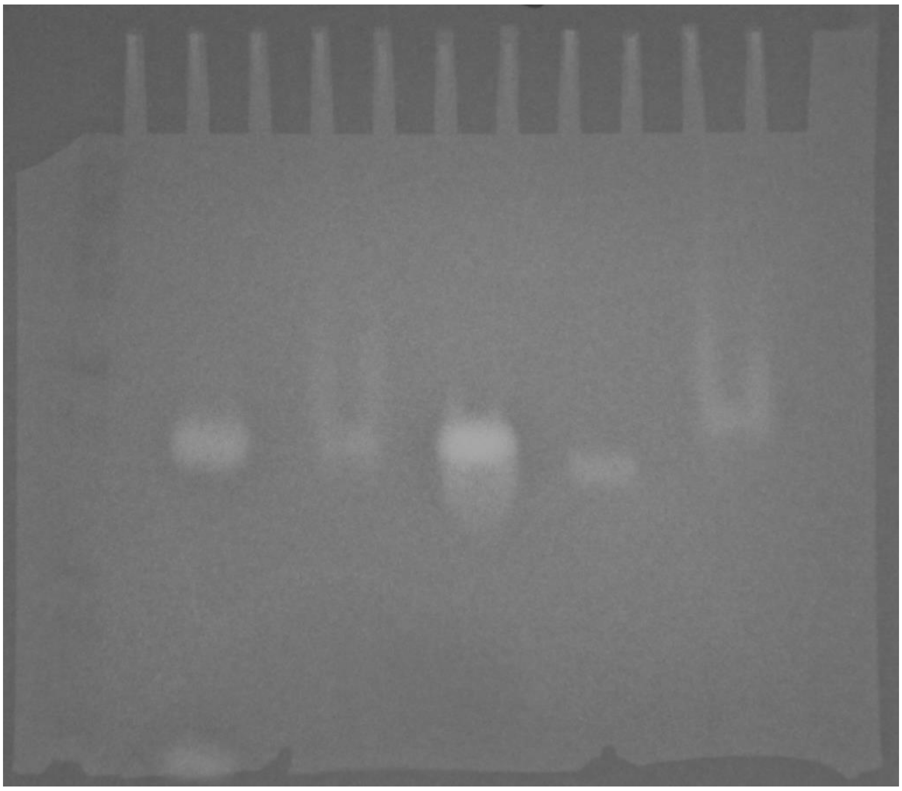

**Supplementary Figure 5.** Unedited SDS Gel overlay displaying zones of inhibition for the production of the bacteriocin ‘equicursorin’ by *L. equicursoris* DSM 19284 (central lane of gel)

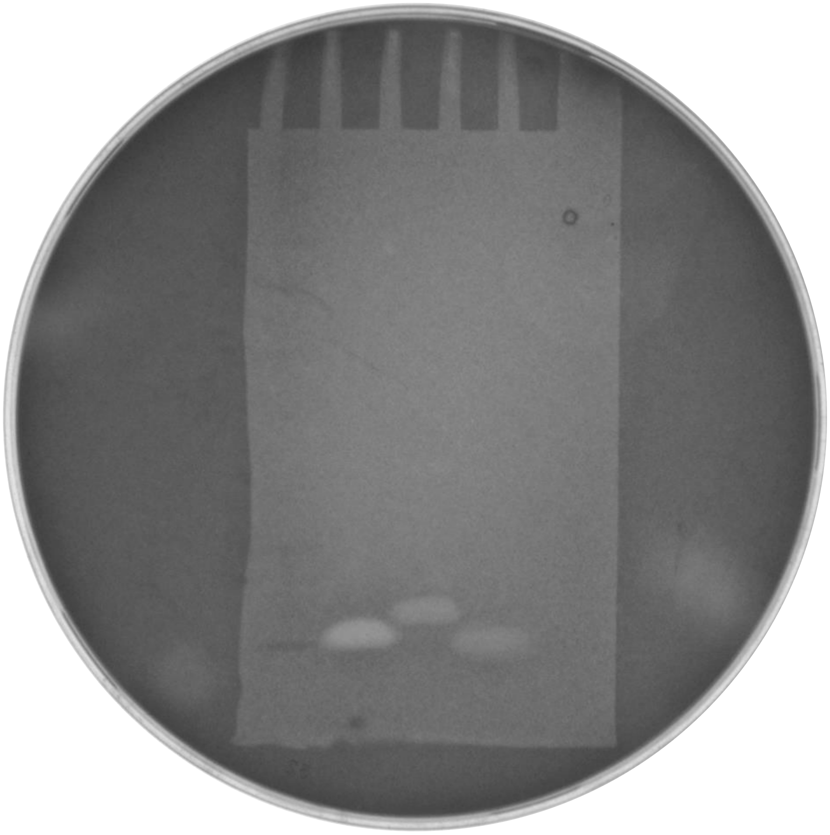

Supplement: Supplementary file 1 — Supplementary Information [file 41598_2017_3339_MOESM1_ESM.pdf]
